# Liquid–liquid phase separation in diseases

**DOI:** 10.1002/mco2.640

**Published:** 2024-07-13

**Authors:** Xinyue Zhang, Lin Yuan, Wanlu Zhang, Yi Zhang, Qun Wu, Chunting Li, Min Wu, Yongye Huang

**Affiliations:** ^1^ College of Life and Health Sciences Northeastern University Shenyang China; ^2^ Laboratory of Research in Parkinson's Disease and Related Disorders Health Sciences Institute China Medical University Shenyang China; ^3^ Department of Pediatrics Ruijin Hospital Affiliated to Shanghai Jiaotong University School of Medicine Shanghai China; ^4^ Wenzhou Institute University of Chinese Academy of Sciences Wenzhou Zhejiang China; ^5^ The Joint Research Center Affiliated Xiangshan Hospital of Wenzhou Medical University Ningbo China; ^6^ Key Laboratory of Bioresource Research and Development of Liaoning Province College of Life and Health Sciences Northeastern University Shenyang China

**Keywords:** cancer, neurodegenerative disease, phase separation, RNA methylation, stress granule

## Abstract

Liquid–liquid phase separation (LLPS), an emerging biophysical phenomenon, can sequester molecules to implement physiological and pathological functions. LLPS implements the assembly of numerous membraneless chambers, including stress granules and P‐bodies, containing RNA and protein. RNA–RNA and RNA–protein interactions play a critical role in LLPS. Scaffolding proteins, through multivalent interactions and external factors, support protein–RNA interaction networks to form condensates involved in a variety of diseases, particularly neurodegenerative diseases and cancer. Modulating LLPS phenomenon in multiple pathogenic proteins for the treatment of neurodegenerative diseases and cancer could present a promising direction, though recent advances in this area are limited. Here, we summarize in detail the complexity of LLPS in constructing signaling pathways and highlight the role of LLPS in neurodegenerative diseases and cancers. We also explore RNA modifications on LLPS to alter diseases progression because these modifications can influence LLPS of certain proteins or the formation of stress granules, and discuss the possibility of proper manipulation of LLPS process to restore cellular homeostasis or develop therapeutic drugs for the eradication of diseases. This review attempts to discuss potential therapeutic opportunities by elaborating on the connection between LLPS, RNA modification, and their roles in diseases.

## Graphical abstract

Liquid–liquid phase separation (LLPS) as a rising molecular entity in the cytoplasm regulates diseases progression via a variety of ways. For example, the relationship between LLPS and cancer can be summarized into six aspects: gene fusions/mutations, heterochromatin formation, transcriptional machinery, cellular homeostasis, microenvironment, and RNA modification. In particular, the behavior and mechanism of LLPS in RNA modification may point to new directions for diagnostics and therapy for some of the most dangerous and hard‐to‐treat tumors.

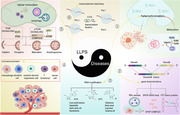

## 1 INTRODUCTION

**FIGURE 1:**
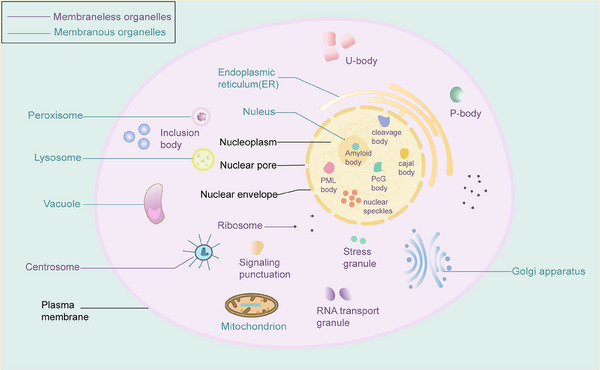
Representative membraneless chambers in eukaryotic cells. Present in the nucleus are mainly gems, cajal body, PML body, nuclear speckles, PcG body, amyloid bodies, and gems. Membraneless compartments, including emergency granules, are located outside the nucleus.

An organelle is a specific subcellular structure, and maintaining organelle homeostasis plays a critical role in inhibiting carcinogenesis and cancer progression. Deregulations have been found in several tumor cell organelles, including mitochondria, endoplasmic reticulum, Golgi apparatus, proteasomes, and lysosomes.^1^ Most common organelles are separated from the internal and external environment by biological membranes, forming sites for specific physiological functions called membranous organelles. There are also a variety of membraneless chambers in cells, including stress granules (SGs), processing bodies (P bodies), promyelocytic leukemia (PML) protein bodies, Balbiani bodies, Cajal bodies, centrosomes, germ granules, heterochromatin, nucleoli, nuclear speckles, paraspeckles, super‐enhancers, and signaling puncta (Figure 1).^2^, ^3^, ^4^ The sophisticated crosstalk between membranous and membraneless organelles is beneficial for maintaining homeostasis. For biomolecules, phase separation exists in the form of liquid droplets.^5^, ^6^ The emerging field of “liquid–liquid” phase separation (LLPS) focuses on the presence, formation, biological functions, and disease associations of membraneless bodies in cells^2^, ^7^ and is associated with cell fate determination, signal transduction, endocytosis, regulation of gene expression and protein translation, and regulation of RNA metabolism.^8^, ^9^, ^10^

Plenty of membraneless chambers are an emerging paradigm of the cellular organization of RNA and RNA‐binding proteins. RNA–RNA interactions and RNA condensation are critical in LLPS, especially for ribonucleoprotein granule formation.^11^, ^12^ Ribonucleoproteins are RNA–protein complexes in prokaryotes and eukaryotes that transport, store, or degrade messenger RNA, thereby indirectly regulating protein synthesis and the more important of these are the SGs.^13^ Various RNA modifications, such as N^6^‐methyladenosine (m^6^A), N^1^‐methyladenosine (m^1^A), and N^7^‐methylguanosine (m^7^G), are involved in LLPS, including but not limited to the formation of SGs and editing of RNA sites.^14^, ^15^, ^16^ Multiple lines of evidence have shown that RNA modification with the m^6^A mark regulates the LLPS and their accumulation in membraneless chambers, such as SGs and P bodies.^17^, ^18^

Various mechanisms function in orchestrating transcriptional and translational regulation to modulate pathological events and therapeutic effects. Among them, m^6^A is the most common transcript chemical modification involved in many pathological processes, including tumorigenesis.^19^ Studies have revealed that m^6^A can dynamically and reversibly regulate all stages of RNA fate by controlling RNA splicing, exportation, degradation, and translation initiation.^20^ Dysregulation of m^6^A on RNAs would lead to the occurrence and development of diseases. Modulating LLPS is an alternative mechanism for m^6^A to maintain RNA stability and control disease progression and therapeutic outcomes.

LLPS is involved in a wide range of diseases, affecting a range of basic cellular pathways, and is most widely engaged in neurodegenerative diseases and cancer. In this review, we introduce the underlying genesis of LLPS in great detail and summarize the role of LLPS in immune signaling pathways, Wnt/β‐Catenin signaling, and other crucial disease‐related signaling pathways. Additionally, the amyloid aggregation has been associated with many neurodegenerative diseases, and aberrant LLPS has been observed in pathologic inclusion bodies in a variety of diseases, including Alzheimer's disease (AD), progressive supranuclear palsy, amyotrophic lateral sclerosis (ALS), and frontotemporal dementia (FTD).^21^, ^22^, ^23^ Cancer‐associated proteins can drive aberrant gene transcription and tumorigenesis through phase separation.^24^ Therefore, this review emphasizes the role of LLPS in neurodegenerative diseases and cancers. Importantly, we would analyze the potential of RNA modifications in regulating LLPS to search for new therapeutic targets and strategies. Approach‐wise, we draft the article with an extensively broad eyesight, from relevant concept to recent progress, from physiological function to pathological implication, from pathogenesis to therapeutic strategy, trying to spark deep thinking and rational analysis of LLPS in the future disease treatment.

## 2 LIQUID–LIQUID PHASE SEPARATION

LLPS transforms weakly interacted macromolecules, such as proteins and RNA, into separate cell droplets. Multiple modular structural domains of functionally similar proteins, intrinsically disordered regions (IDRs) of proteins (Table 1), and RNAs mediate multivalent interactions within and between biomolecules.^25^ Individual droplets can move freely in the solution, gradually collide, and fuse to form a bigger‐sized structural entity.^26^ The main driver of LLPS may involve multivalent interactions. Molecules with multivalent interactions can form aggregates that are constantly enriched with each other, leading to an increment in the concentration of molecules in the aggregates. Until the solubility limit, aggregates would precipitate from the solution as LLPS. Various factors, including concentration and phosphorylation, contribute to LLPS, and different triggers determine different fates and physiological functions.^27^ Physical modulators like temperature and pressure can also regulate LLPS behavior. Scaffold protein theory uses scaffold proteins as driver molecules to form a protein–RNA network to drive the formation of phase separation.^28^ Two protein types are involved in facilitating the formation of such interaction networks: one class is characterized by multiple folded structural domains, such as the SH3 structural domain in Nck proteins, where interactions between the SH3 structural domains in Nck and proline‐rich motifs (PRMs) in N‐WASPs assemble into higher‐order oligomers; another class of proteins is characterized by IDR.^29^ IDR‐containing proteins are enriched in many condensates and have different phase transition capabilities depending on the length and number of IDR and the characteristics of the IDR sequences.^24^ A common feature of IDRs is that they consist of low‐complexity sequence regions (LCRs), such as repeated sequences of a single amino acid.^30^ In summary, the two proteins have similarities and interact through multiple structural domains or modules.

**TABLE 1:** Prediction of protein disorder structure for major human cancer related genes.

Genes (abbreviation)	Number residues disordered	Disordered/predicted residues (%)	Function
Tumor protein 53 (P53)	193	49.11	Encoding tumor suppressor proteins containing transcriptional activation, DNA binding, and oligomerization domains
Retinoblastoma (Rb)	356	38.36	Negative regulator of the cell cycle
Adenomatous polyposis coli (APC)	1838	64.65	It encodes a tumor suppressor protein that acts as an antagonist of the Wnt signaling pathway.
Nonmetastatic protein 23 (NM23)	22	12.22	A housekeeping enzyme and mainly considered as a tumor suppressor gene
Neurofibromin 1 (NF‐1)	547	21.86	Function as a negative regulator of the Ras signal transduction pathway
Synaptotagmin binding cytoplasmic RNA interacting protein (SYNCRIP)	268	43.02	Member of the cellular heterogeneous nuclear ribonucleoprotein family
G3BP stress granule assembly factor 1 (G3BP1)	10	9.52	DNA‐unwinding enzyme prefers partially unwound 3′‐tailed substrates and can also unwind partial RNA/DNA and RNA/RNA duplexes in an ATP‐dependent fashion.
DEAD‐box helicase 3 X‐linked(DDX3X)	97	20.42	Member of the DEAD‐box protein family
YTH N^6^‐methyladenosine RNA binding protein F2 (YTHDF2)	246	46.5	Member of the YTH (YT521‐B homology) superfamily
Ataxin 2 like (ATXN2L)	351	87.53	The Nod1/Apaf‐1 family members encode a protein with two caspase recruitment (CARD) domains and six leucine‐rich repeats (LRRs)
Argonaute2 (AGO2)	104	12.61	Member of the Argonaute family of proteins that play a role in RNA interference
Insulin like growth factor 2 mRNA binding protein 1 (IGF2BP1)	157	19.03	Similar to insulin in function and structure, it is a member of a family of proteins involved in mediating growth and development.
Mov10 RNA helicase (MOV10)	158	15.75	Enable RNA helicase activity and RNA binding activity to participate in the defense response to viruses; negative regulation of translocation, RNA‐mediated; posttranscriptional regulation of gene expression
Cell cycle‐associated protein 1 (CAPRIN1)	390	62.10	Produces several transcriptional variants that act as CDK4 kinase inhibitors
G3BP stress granule assembly factor 2 (G3BP2)	84	42.42	DNA‐unwinding enzyme that prefers partially unwound 3′‐tailed substrates and can also unwind partial RNA/DNA and RNA/RNA duplexes in an ATP‐dependent fashion
FMR1 autosomal homolog 1 (FXR1)	338	55.59	An RNA binding protein that interacts with the functionally similar proteins FMR1 and FXR2
FMR1 autosomal homolog 2 (FXR2)	428	63.60	An RNA‐binding protein containing two KH domains and a RCG box, which is associated with polysomes
UPF1 RNA helicase and ATPase (UPF1)	349	30.91	Part of a postsplicing multiprotein complex involved in both mRNA nuclear export and mRNA surveillance
Poly(A) binding protein cytoplasmic 4 (PABPC4)	294	44.48	Poly(A)‐binding proteins (PABPs) bind to the poly(A) tail present at the 3‐prime ends of most eukaryotic mRNAs.
Ubiquitin specific peptidase 10 (USP10)	194	36	Member of the ubiquitin‐specific protease family of cysteine proteases

In cells, LLPS triggers the formation of membraneless chambers. Membraneless chambers are involved in cellular physiological responses through specific proteins and RNAs within them. In addition, membraneless chambers facilitate gene regulation through different mechanisms and play critical roles in a variety of biological processes, including RNA metabolism, translation, protein modification, and signal transduction.^31^, ^32^ LLPS, an organizational pattern of biological macromolecules, is essential for signal transduction and regulation of gene expression. Once the formation or dynamics of normal LLPS is changed, it can lead to the development of diseases.^33^ A typical characteristic of LLPS is a saturation concentration, which means the LLPS structure is formed only when the concentration of components exceeds a given threshold. LLPS structures can exhibit different properties under different factors and environmental influences, such as salt concentration, temperature, and other ions. Material properties, such as viscoelastic liquids, network fluids, liquid‐crystalline, micellar, or semi/para‐crystalline condensates, have been proposed to be risk factors in several diseases.^34^ In Parkinson's disease (PD), tau proteins may act as scaffolding proteins for neuronal cohesion, and full‐length but not carboxy‐terminally truncated α‐synuclein is concentrated within tau droplets under the regulation of tau phosphorylation.^35^, ^36^ In summary, scaffold molecules constitute the driving factor for phase separation and occur under specific physicochemical properties. At the same time, modifications, such as phosphorylation, sumoylation, ubiquitination, and methylation, can alter the course and extent of LLPS.

There are two sides, good and bad, to any matter, and phase‐separated structures play different roles in various biological processes and the pathogenesis of protein aggregation diseases. From the beneficial side, LLPS can locally concentrate molecules in the condensate to activate cytoskeletal structural responses, signaling processes, nucleation and RNA processing reactions. For example, miRNA‐induced silencing complex (miRISC) is a multiprotein assembly in which microRNAs (miRNAs) recognize target‐repressed mRNA and functions in an LLPS‐dependent manner. Argonaute2 (Ago2) and TNRC6B, the two core protein components of miRISC, isolate target RNAs from bulk solution in living cells via structural domains separated by multivalent interactions with tryptophan and condense miRISC droplets to recruit a deadenylation factor.^37^ Ago2 and TNRC6B condensed into phase‐separated droplets are essential for mRNA translation and stability in eukaryotes. miRISC is a multiprotein assembly that uses microRNAs to recognize mRNAs targeted for repression.^38^ LLPS is also manifested in a variety of diseases.^39^, ^40^ Protein aggregation is a significant cause of many neurodegenerative diseases, attacking the brain and killing neurons. These proteins (e.g., α‐synuclein, FUS, tau, and TDP‐43) undergo LLPS independently through electrostatic interactions, increasing protein concentration. This process of molecular self‐assembly is present in a variety of pathological inclusions.^41^, ^42^, ^43^ miRISC might implicate cancer initiation and progression in specific cellular contexts. Aberrant formation and regulation of LLPS can lead to malignant transformations to acquire cancer hallmarks, such as evading growth suppressors, resisting cell death, sustaining proliferative signals, and inducing genome instability.^3^, ^4^ Plenty of proteins with IDR regions have irreplaceable roles in multiple signaling pathways,^44^, ^45^, ^46^ and their overexpression, mutation, or fusion can lead to under‐ or overactivation of pathways.

## 3 LLPS IS AN IMPORTANT HUB IN SEVERAL SIGNALING PATHWAYS

Studies have demonstrated the role of LLPS in the immune response, not limited to immune cell maturation and activation, immune signaling, and immunomodulation.^47^, ^48^ In addition, several important signaling pathways functioned in the accompanied with LLPS.

### 3.1 T‐cell receptor and B‐cell receptor immune signaling

Immune signaling pathways alter the conformation of immune receptors by ligand binding of pathogenic stimuli, inducing spatial reorganization and, thus information transfer. LLPS is found in a variety of immune signaling pathways, including the T‐cell receptor (TCR)^49^ and the B‐cell receptor (BCR)^50^ pathway, as well as cytoplasmic signaling pathways, such as cGAS–STING.^51^ Normally, LLPS is recognized as 3D structures formed in the cytoplasm and nucleus, but 2D membrane‐associated condensates formed along the cell membrane are well represented in immune signaling.^52^ Membrane LLPS restricts protein movement to higher concentrations and induces a lower protein concentration threshold in condensates than in membraneless organelles.^53^ On the surface of the cell membrane of immune cells, there are membrane clusters^54^ that LLPS regulates and thus proceed the immune signaling cascade.^55^, ^56^ To decipher how LLPS drives the formation of immune signaling condensates to establish immunity could contribute to developmental research and disease treatment.

The TCR signaling pathway depends on T cell microclusters on the plasma membrane,^57^ which contain multiple transmembrane receptors.^58^ Components of clusters typically have high densities and low mobilities. High densities allow more frequent contact between molecules within the cluster and low mobilities mean less time for interactions to occur.^59^ In addition to the effects of microclusters, the combined effects between proteins and lipids drive LLPS between TCR‐associated proteins in the proximal part of the membrane. For example, cholesterol enhances protein condensate TCR clustering, whereas downstream TCR triggering TCR clustering promotes cholesterol LLPS.^60^, ^61^ It has been shown that LAT, GRB2, and SOS1 can form oligomers through multivalent interactions.^62^ In the signal transduction system of the T‐cell junction protein LAT, stimulation induces phosphorylation of specific residues to generate multiple binding sites to promote LLPS of the signal transduction protein.^49^, ^63^, ^64^ Transmembrane proteins and their cytoplasmic binding partners (GRB2 of LAT) induce LLPS, which in turn prompts the multivalent SH3 structural domain‐PRM to bind to a downstream binding partner (SOS1 of GRB2).^55^, ^65^ In addition, the dependence of LLPS on phosphorylation by harmful feedback regulatory mechanisms means that it will be significantly reduced or eliminated without a stimulus.^66^ LAT condensate promotes tyrosine phosphorylation and marks the activation of the TCR signaling pathway. However, LAT can be bypassed in the microcluster formation of chimeric antigen receptors (CARs) by establishing multivalent interactions between CARs and the LAT‐binding chaperone GADS.^67^ Distinct physical and biochemical compartments LLPS create can facilitate different TCR signaling.

**FIGURE 2:**
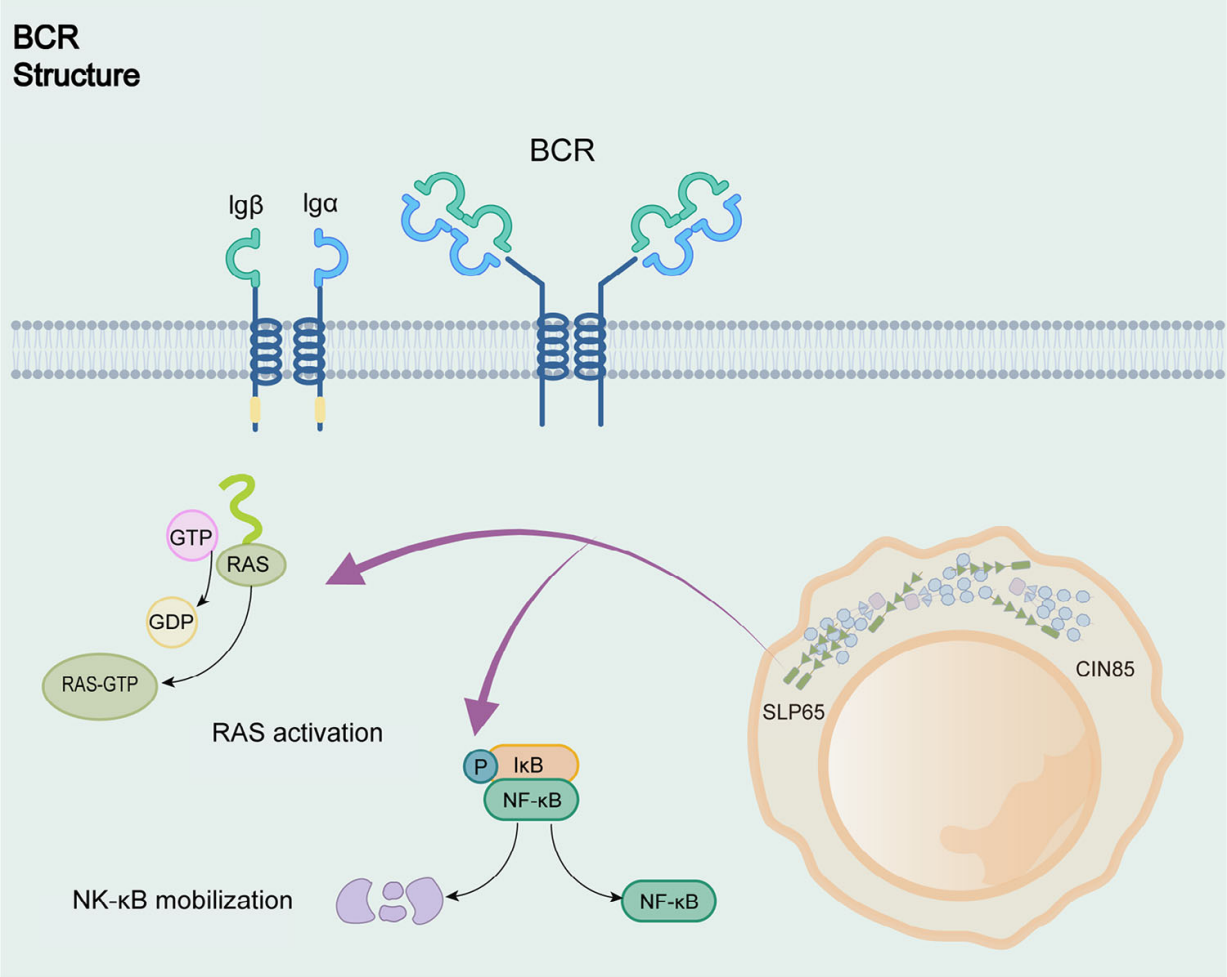
Signal transduction condenses in the B cell receptor pathway. The scaffolding protein SLP65 and its binding chaperone CIN85 form liquid condensates by multivalent interactions in B cells. The proximity of condensate to the plasma membrane further triggers RAS activation and NF‐κB mobilization.

The scaffolding protein SLP65 (also known as BLNK) possesses a critical function in the BCR signaling pathway in driving LLPS, similar to LAT in the TCR signaling pathway (Figure 2). SLP65 with its binding partner CIN85, undergoes LLPS via multivalent interactions between the SH3 structural domain of CIN85 and the proline motifs of SLP65.^68^ Liposomes play a crucial role in facilitating cohesion formation. SLP65 contains an amino‐terminal lipid‐binding domain that binds to small, highly curved vesicles.^50^ SLP65 mutants lacking the N‐terminal vesicle‐binding domain fail to undergo LLPS at the cytoplasm or plasma membrane, leading to defects in BCR signaling, such as calcium inward flow.^50^ Higher‐order assembly provides a theme in sensitivity control, signal transduction, innate immunity and adaptive. BCR downstream has a ternary complex called CBM signalosome, generated by CARM1, BCL10, and MALT1 assembly, which mediates NF‐κB activation.^69^, ^70^, ^71^ These higher‐order assemblies with solid‐like behaviors or phase‐separated liquid‐like droplets can inhibit unnecessary immune activation and enable activation of proximity‐driven protein and spatial control of immune signaling.

### 3.2 cGAS–STING signaling

In response to stimulation by pathogens or damaged cytoplasmic dsDNA, cGAS would be activated and binds to dsDNA.^72^ Cyclic GMP–AMP (cGAMP) synthesizes and activates STING, which transduces downstream signals and induces the expression of proinflammatory cytokines.^73^ Sensing DNA, cGAS–STING signaling triggers innate immune responses and is an important axis of autoimmunity, sterile inflammatory response and cellular senescence.^74^ Besides the role of immune defense, the GAS–STING DNA‐sensing pathway has also been shown to be connected with antitumor immunity, exhibiting paradoxical function in both immune surveillance for tumor inhibition and immune‐suppressive tumor microenvironment for metastasis‐promoting.

cGAS consists of a positively charged disordered N‐terminal and a structured C‐terminal region. Depending on the positively charged residue at the N‐terminal and the DNA‐binding site in the C‐terminal, cGAS can induce LLPS upon DNA binding.^48^, ^75^, ^76^ Long DNA strands, free zinc ions and DNA‐binding structural domains in the catalytic core of cGAS are significant factors contributing to LLPS formation.^77^ Factors affecting the occurrence of LLPS in cGAS have now been found to be multiple.^51^, ^78^ It has been reported that the binding of streptavidin to cGAS enhances the interaction of cGAS with DNA and thus promotes the formation of its condensation complex.^79^ However, ORF52/VP22‐type tegument proteins, as a family of inhibitors against cGAS–DNA phase separation, restrict cGAS–DNA LLPS. ORF52/VP22‐mediated restriction of cGAS–DNA LLPS is implemented by self‐condensation with viral genomic DNA without strong direct interaction between viral proteins and cGAS.^80^ This method of altering one's structure to undergo LLPS with DNA to inhibit phase‐separated polymers cleverly evades head‐on counteracting cGAS–STING immunization. Also, it demonstrates the impact of LLPS on the way biomolecules interact with each other. It has been shown that the percentage of cells containing cGAS condensates is significantly lower in G3BP1‐deficient cells^81^, ^82^; however, the underlying mechanism remains obscure. LLPS could also modulate cGAS–STING cascades. For example, LLPS of the oncogene neurofibromin 2 (NF2) mutant inhibits the cGAS–STING pathway.^83^ NF2 promotes innate immune‐sensing of cytoplasmic nucleic acids and has a missense mutant NF2m. NF2m aggregates into dynamic condensates in the cytoplasm induced by IRF3 in its activated state. Aggregates of NF2m can restore average tumor growth in a mouse model of STING‐initiated antitumor immunity and hinder STING‐initiated antitumor immunity.

### 3.3 Wnt/β‐Catenin signaling

The Wnt/β‐Catenin pathway regulates the cellular transcriptional program and plays a vital role in the organism's homeostasis and development.^84^, ^85^ APC and Axin are key multidomain scaffolding proteins in the signaling pathway. The G protein signaling domain of Axin has an intrinsically disordered intermediate region that contains multiple kinases and APC binding sites.^50^, ^86^, ^87^ When Axin is expressed in different cell types, it spreads into a “dot” to increase its concentration, recruiting APCs and other destructive complex proteins.^88^, ^89^ There is evidence that the complex controlling Wnt/β‐catenin is formed precisely by the protein LLPS driven by Axin.^90^ The complex facilitates phosphorylation of β‐catenin by GSK3β, which is essential for the regulation of β‐catenin protein stability to maintain Wnt/β‐catenin signaling.^91^, ^92^ The β‐catenin destruction complex and its receptor signalosome can robustly and precisely regulate Wnt/β‐catenin signaling. Assembly of Wnt signalosome is driven by Dishevelled and Axin copolymerization.^93^, ^94^ Dishvelled‐2 (Dvl2) has an IDR at the N‐terminus and can undergo LLPS in vitro and cells.^95^, ^96^ The formation of Dvl2 condensates depend on ubiquitylation of Dvl2 through K63 linkage by WWP2.^95^ LLPS of Dvl2 has been found to be facilitated by signalosome components (e.g., Fzd5).^96^ In addition, Dvl2 LLPS weakens LLPS of Axin that is organized into the signalosome and displays high mobility at low concentration.^96^ Wnt signalosome and phase‐separated β‐catenin destruction complex emphasize the distinct mechanism LLPS provides in signal transduction.

### 3.4 RAS/MAPK signaling

The RAS/MAPK signaling pathway is involved in a variety of cellular processes, including differentiation, proliferation, and survival, and their mutations occur frequently in many tumors making it one of the most critical pathways associated with cancer.^97^, ^98^, ^99^ The nonreceptor protein tyrosine phosphatase SHP2 is crucial in RAS–MAPK signaling during normal development.^100^ The NH2‐terminal end of SHP2 has two tandem SH2 structural domains, a central PTP‐catalyzed structural domain, and a C‐terminal tail.^101^ SHP2’ folded PTP structural domain mediates its LLPS by intermolecular electrostatic interactions.^102^ Wild‐type SHP2 LLPS could be recruited and promoted by disease‐related mutation of SHP2 to induce MAPK hyperactivation.^102^ In another study, ERK signal responsive repressor ERF has been determined to form condensates vis LLPS, and ERF condensates can be attenuated using ERK inhibitor U0126.^103^ Generally, some components of RAS/MAPK signaling pathway display LLPS behaviors, and RAS/MAPK signaling could also participate in the modulation of LLPS to adjust cellular organization.

### 3.5 Hippo/YAP signaling

The Hippo–YAP signaling network integrates signals with its transcription factor YAP protein to control cell proliferation and differentiation.^104^, ^105^ Activation of this pathway promotes regeneration of damaged organs and is associated with tumorigenesis.^106^, ^107^, ^108^ Several components of Hippo–YAP signaling include low‐complexity domains and can undergo LLPS. Hippo signaling complexes undergoing LLPS to produce biomolecular condensates in the cytoplasm has been documented.^109^ LLPS of the Hippo–YAP signaling pathway is closely related to super‐enhancers. YAP LLPS occurs near chromatin compartmentalizes YAP from other relevant coactivators to induce transcription of YAP‐specific proliferative genes.^110^ YAP coalescence occurs in the super‐enhancer region, regulates cell proliferation and survival, and activates target genes.^111^ It has been shown that hyperactivated YAP promotes super‐enhancer condensates in embryonic stem cells through LLPS, leading to gene‐specific and restrictive regulation to control lineage differentiation.^112^ Glucose in tumor cells tends to be consumed in increased amounts to support tumor proliferation, and it has been found that accumulated glycogen can undergo LLPS.^113^ Glycogen droplets segregate Hippo kinase from YAP so that inhibition of the latter is deregulated, the Hippo pathway is inactivated, and the activity of the downstream proto‐oncoprotein YAP is increased, ultimately promoting tumor growth.^114^, ^115^ Destroying YAP LLPS prevents tumor growth, increases immune response, and enhances the sensitivity of anti‐PD‐1 therapy,^116^ indicating that YAP LLPS can be applied as a therapeutic target for anti‐PD‐1 therapy. In addition, DDR1, a collagen‐binding receptor tyrosine kinase, has been found to counteract the Hippo/YAP pathway in a LLPS‐dependent manner.^117^ Therefore, LLPS can regulate the Hippo/YAP pathway and can also be controlled by the Hippo/YAP pathway to exert different functions.

## 4 LLPS PHENOMENON IN NEURODEGENERATIVE DISEASES

Neurodegenerative diseases tend to have one thing in common: protein aggregates. These aggregated proteins have a propensity for LLPS.^42^ Currently, some studies have now observed the link between LLPS and neurodegenerative diseases, including ALS,^118^, ^119^ AD, and some forms of FTD.^120^, ^121^, ^122^ Here, we discuss the LLPS of four major proteins in neurodegenerative diseases: α‐synuclein, fused in sarcoma (FUS), tau, and TAR DNA‐binding protein 43 (TDP‐43).

### 4.1 TAR DNA‐binding protein 43

As a significant nuclear RNA/DNA‐binding protein with a prion‐like structural domain, TDP‐43 regulates various RNA processing steps. It accumulates in the cytoplasm of patients with a variety of neurodegenerative diseases.^123^, ^124^ TDP‐43 consists mainly of two RNA recognition motifs, a structured N‐terminal domain with a C‐terminal glycine‐rich low‐complexity structural domain.^125^, ^126^ Recognition motifs of TDP‐43 are primarily involved in RNA translocation, stability, and splicing,^127^ whereas the disordered C‐terminal structural domains^126^, ^128^, ^129^ with highly conserved N‐terminal structural domains^130^ are connected to LLPS. TDP‐43 can undergo LLPS, and liquid phase‐separated droplets containing TDP‐43 can be converted into gel/solid structures under prolonged stress and even to amyloid fibril structures in vitro.^131^ In normal cells, phase separation of these RNA‐binding proteins is ordered and controlled, with TDP‐43 acting as a liquid shell and encapsulating the HSP70 family chaperone.^132^ As molecular chaperones, the HSP70 family maintains proteostasis to protect proteins from misfolding and aggregation^133^, ^134^ and is reported to be transcriptionally downregulated in AD.^135^ Furthermore, under oxidative stress, HSPB1, along with HSP70 chaperone activity, is central to the maintenance of TDP‐43 protein homeostasis through the interaction of the low‐complexity structural domains with TDP‐43.^136^ Similar to the C‐terminal domain, N‐terminal domain interactions are also important for cellular function. In addition to ensuring that the expansion of the aggregation‐prone region of the C‐terminal structural domain drives endogenous TDP‐43 aggregates and sequesters,^137^ LLPS is also affected by the expansion of the aggregation‐prone region of the structural domain. Wang et al.^130^ reported that disrupted TDP‐43 N‐terminal domain polymer by phosphomimetic substitution at S48 can block LLPS and disrupt RNA splicing activity in vitro.

### 4.2 Fused in sarcoma

Similar to TP‐43, FUS is a widely expressed RNA‐binding protein involved in multiple RNA metabolic pathways.^130^, ^138^, ^139^, ^140^ The N‐terminal domain of FUS is a highly conserved low‐complexity structural domain that mediates protein–protein interactions and drives the aggregation of FUS into protein inclusion bodies. This region can undergo reversible LLPS.^141^, ^142^ Furthermore, it has been shown that cation–π interactions between tyrosine in the low‐complexity structural domain and arginine in the structured C‐terminal structural domain promote LLPS and gelation under the regulation of methylation.^143^ ALS is the most common motor neuron disease in adults, and cytoplasmic mislocalization and aggregation of FUS in neurons and glial cells in affected individuals is the cause of ALS.^144^, ^145^ Transient SGs are formed when neurons encounter cellular stress, sequestering untranslated mRNAs and associated proteins to reduce energy requirements.^146^ Overexpression of FUS induces the spontaneous formation of SGs and is recruited to this membraneless organelle.^147^ The persistence of SGs provides an environment for mutations in RNA‐binding proteins such as FUS,^148^, ^149^ and SGs may be a locus for disease biogenesis.^43^ Also, there may be transmission factors in diseases such as ALS/FTD. It has been found that prion‐like mechanisms in neurodegenerative diseases can transmit protein misfolding and aggregation.^150^ TDP‐43 and FUS in ALS have low‐complexity structural domains similar in amino acid composition to yeast PrLDs (prion‐like domains; PrLDs).^151^ Prion‐like propagation mechanisms are active in ALS and FTD, and preformed recombinant TDP‐43 fibers in neuronal cell lines can trigger overexpression and aggregation of endogenous TDP‐43.^152^, ^153^

### 4.3 Tau

Tau consists of four distinct regions: the N‐terminal projection domains, the proline‐rich domains, the microtubule‐binding domains, and the C‐terminal domains.^41^ This structure facilitates binding to microtubules and allows for an intrinsically disordered and inhomogeneous charge distribution over the entire length of the tau.^154^, ^155^ Tau is a soluble neuron‐specific microtubule‐binding protein that is a significant component of inclusion bodies in various neurodegenerative diseases, including AD and FTD.^22^, ^156^, ^157^ Currently, one critical factor for the proteotoxicity of tau is the formation of neurofibrillary tangles, the abundance of which correlates with disease progression.^22^, ^157^ Proteins are susceptible to a large number of posttranslational modifications such as phosphorylation, acetylation, and ubiquitination, which are involved in the pathogenesis of neurodegenerative disorders.^158^ neurons can release and take up hyperphosphorylated tau, triggering templated tau misfolding in neurons, leading to cytotoxicity.^159^ It has been repeatedly observed that tau forms droplets via LLPS under physiological conditions, regulates microtubule assembly and function, and may be converted to amyloid aggregates upon aging.^160^, ^161^ It is currently believed that tau undergoes LLPS driven by attractive intermolecular electrostatic interactions between the negatively charged N‐terminal and positively charged middle/C‐terminal structural domains of the protein.^160^, ^162^ Also, aberrant LLPS may occur when tau binds to other molecules. For example, tau undergoes complex coalescence after binding to RNA.^163^, ^164^

### 4.4 α‐Synuclein

α‐Synuclein is structurally and functionally highly distinct from TDP‐43, FUS, and Tau and is intimately associated with Lewy body formation.^165^, ^166^ Lewy bodies are a characteristic hallmark of PD, leading to neuronal death.^167^ α‐Synuclein consists of a membrane‐bound structural domain consisting of a positively charged N‐terminal structural domain, a hydrophobic nonamyloid component, and an acidic structural domain containing the C‐terminal structural domain, which is involved in its controversial nuclear localization and various interactions.^168^, ^169^ The C‐terminal region of α‐synuclein undergoes long‐range interactions with the N‐terminal region and can remain monomeric, highly flexible, and disordered after the occurrence of LLPS.^170^ The α‐synuclein LLPS is driven by electrostatic interactions in the amphiphilic N‐terminal structural domain and hydrophobic interactions between nonamyloid components.^171^ High concentrations of α‐synuclein trigger the process of LLPS and the formation of amyloid hydrogels containing oligomeric and fibrillar material.^171^, ^172^ Unstructured proteins of α‐synuclein form partially folded intermediates, then oligomerize and protofibrillate, ultimately giving rise to amyloids.^173^, ^174^ Furthermore, it has been shown that abnormally aggregated α‐synuclein molecules induce normal molecular misfolding to form polymers and are taken up by neurons, leading to neurodegenerative alternations.^175^ α‐Synuclein aggregates are also closely related to mitochondrial autophagy. Aggregates interfere with the normal function of mitochondrial autophagy‐associated proteins, thereby affecting mitochondrial autophagy processes.^176^

## 5 LLPS IN CARCINOGENESIS AND CANCER DEVELOPMENT

Cancer has traditionally been regarded as a genetic illness, and subsequently, epigenetic abnormalities and tumor microenvironment are all found to be critical for tumorigenesis.^177^, ^178^ Genetic alterations in dysregulated transcriptional programs contribute to cancer development, and modulation of gene expression may also help improve the outcome of cancer treatment. Gene expression is a biological process in which genetic information guides the synthesis of RNA molecules that code for proteins or build noncoding RNAs (ncRNAs), and any failure in its overall expression, such as abnormal protein expression and epigenetic alterations, may promote the proliferation and metastasis of cancer cells. Various mechanisms orchestrate transcriptional and translational regulation to modulate tumorigenesis events and therapeutic effects. m^6^A is the most prevalent transcript chemical modification and is involved in many pathological processes, including tumorigenesis.^19^ Studies have revealed that m^6^A can dynamically and reversibly regulate all stages of RNA fate via controlling RNA splicing, exportation, degradation, and translation initiation.^20^ Exploiting the role of transcriptional machinery and posttranscriptional network in cancer occurrence and therapy has the potential for searching for new therapeutic targets and strategies.

Abnormal gene expression profiles, or dysregulation of oncogene or tumor suppressor gene activity following degradation of proteins required for average cell growth, drive many abnormal responses, such as aberrant gene amplification, missense mutations, malfunctioning chromosomal translocations, cellular conditions and external signals, and cell acquiring uncontrolled proliferation.^179^ LLPS of cancer‐related proteins involved in epigenetic and transcriptional regulation, translation, signal transduction, and protein degradation plays a crucial role in cancer development (Table 2).^180^ Generally, LLPS might participate in all cancer biological events, including tumorigenesis, cancer development and therapy.

**TABLE 2:** Summary of cancer‐related proteins involved in LLPS.

Dysregulated process	Protein	Impact of LLPS on cancer development (reference)
Signal transduction	T cell receptor (TCR)	Cancer‐associated antigens; tumor immune mediators^181^, ^182^
	Beta‐catenin	Mutated Wnt pathways lead to a variety of growth‐related pathologies and cancers.^93^
	Zona occludens (ZO)	Fusion of dense protein polymerization and linker bands are driven by LLPS transitions.^183^
	Tumor protein p53 binding protein 1 (TP53BP1)	53BP1 LLPS increases p53 target gene expression and repairs DNA damage.^184^
	Son of sevenless (SOS)	Activation of Ras, a cofactor involved in RAS signaling in tumor development^185^
Epigenetics	Heterochromatin protein 1 (HP1α)	LLPS leads to gene silencing of HP1 in heterochromatin.^186^
	Bromodomain‐containing protein 4 (BRD4)	LLPS‐mediated transcriptional dysregulation renders cancer cells highly dependent on the transcriptional regulator BRD4.^187^
	Chromobox protein homolog2(CBX2)	IDR of LLPS‐regulated cbx2 is involved in chromatin regulation of testis development.^188^
	Heat shock factor 1 (HSF1)	LLPS segregation downregulates HSF1 function, and chaperone gene induction is reduced.^189^, ^190^
Transcription	m^6^A‐related proteins	Modulation of RNA transcripts through LLPS‐mediated deregulation, thereby affecting tumorigenesis^191^
	Yes1 associated transcriptional regulator/WW domain‐containing transcription regulator 1 (YAP/TAZ)	TAZ increases activity in various cancers by forming nuclear condensates through LLPS.^189^
	Octamer‐binding transcription factor 4(OCT4)	Mediator enhances LLPS, activates genes, and controls gene transcription.^192^
	Heterogeneous ribonucleoprotein particle	LLPS improves transcriptional response and synergizes chromosomal enhancer assembly in breast cancer.^193^
	Mediator complex subunit 1 (MED1)	Coactivators overexpressed and modified in cancer^194^, ^195^
	Polypyrimidine tract‐binding protein 1 (PBP1)	LLPS promotes p62 recruitment and Nrf2‐mediated stress response.^196^
Protein degradation	C/EBP homologous protein (CHOP)	LLPS degradation of carcinogenic substrates^197^
	Fused in sarcoma (FUS)	Recruitment of DNA damage sites, assembly of damaged DNA‐rich compartments, transcriptional coupling repair effectors^198^
RNA repair	RAD52 homolog, DNA repair protein (RAD52)	LLPS induces the formation of DNA double‐strand breaks and colocalization with Rad52, which increases the mobility of damaged chromatin.^199^

### 5.1 LLPS contributes to aberrant transformation via gene fusion and mutation

Disordered structures of various proteins form droplets through LLPS, repeated fusions among genes that bind proteins, and chromosomal translocations that produce multiple chimeras.^200^ SPOP–DAXX bodies, PML vesicles, and FET fusion proteins formed via multivalent interactions affect various cancers regarding gene fusion and mutation.

#### 5.1.1 SPOP–DAXX body

Spotted POZ (poxvirus and zinc finger protein) protein (SPOP), a broad complex, tram‐track and small curio (BTB) complex protein, acts as a substrate articulator for Cullin 3 RING E3 ubiquitin ligases to target various proto‐oncoproteins for ubiquitination and their proteasomal degradation.^201^ Normally, SPOP assembles with substrates and recruits to the nucleosome, but cancer‐associated mutations disrupt the SPOP/substrate colocalization mechanism and substrate‐mediated LLPS of ubiquitin ligases. Substrates are candidates that mediate separation from SPOP and regulate its subcellular localization and function, including death domain‐associated proteins (DAXX), androgen receptors (AR), and other critical signaling cascade effectors.^202^, ^203^ DAXX has potent oncogenic properties and regulates multiple processes, including transcription, DNA damage response, cell signaling and cell death.^204^ The SPOP‐mediated degradation of DAXX induces apoptosis and extracellular matrix degradation.^205^ Thus, SPOP mutants disrupt substrate binding and LLPS, resulting in the inability to colocalize and flip with the substrate.^197^ Disruption of interaction among substrates by SPOP mutations may inhibit tumor formation through uncontrolled DAXX ubiquitination activity.

#### 5.1.2 PML vesicles

PML vesicles, 0.1–1.0 µm in diameter, are membraneless nuclear substructures that function as tumor suppressors via regulating cell cycle, senescence, programmed cell death and DNA damage response.^206^, ^207^ PML vesicles are biomolecular condensates assembled by LLPS of members of a protein superfamily containing a tripartite motif and identified initially as fusion partners with the retinoic acid receptor alpha (RARα) generated by the t(15;17) genomic translocation in acute promyelocytic leukemia (APL).^208^ Both nuclear receptor‐induced differentiation and PML‐triggered apoptosis can be interfered with PML–RARα proteins. The loss of function of PML and RARα is implicated in APL pathogenicity, and inactivation of the tumor suppressor activity of PML might promote cancer development by inducing genome instability.

#### 5.1.3 FET fusion proteins (EWS–FLI, FUS–CHOP)

FUS, Ewing sarcoma breakpoint region 1 (EWSR1, also known as EWS), and TBP‐associated factor 15 (TAF15) make up FET (FUS/EWSR1/TAF15) RNA‐binding protein family, which is involved in multiple steps of RNA metabolism, including transcription, RNA processing, and the cytoplasmic fates of mRNAs.^209^, ^210^ FUS participates in RNA transport, splicing and translation and has been shown to undergo LLPS to form granules in various types of cells.^211^ CHOP (DDIT3/GADD153), a transcription factor family member, can be upregulated by ER stress, growth arrest, and DNA damage.^142^, ^212^ FUS–CHOP fusion oncoprotein is a genetic hallmark of the myxoid/round cell type and promotes myxoid liposarcoma tumorigenesis and metastasis.^213^, ^214^ EWSR1 is a multifunctional RNA‐binding protein often fused to various partner genes due to chromosomal translocation. EWSR1–Friend leukemia integration one transcription factor (FLI1) acts as a potent tumor‐specific chimeric oncoprotein, and high EWSR1–FLI1 expression exponentially endows cancer cell proliferation.^215^ EWSR1–FLI1 fusion protein is found in approximately 85% of sarcomas and is required for Ewing's sarcoma tumorigenicity.^216^, ^217^ FUS–CHOP and EWSR1–FLI1 are highly localized to the nucleus and drive aberrant transcriptional programs in Ewing's and liposarcoma, respectively.^218^

LLPS aggregates FUS and EWSR1 to enhance transcriptional activity.^219^ FUS has been shown to undergo LLPS both in vivo and in vitro to generate functional RNA granules and aggregates. In addition, the LLPS of FUS can be enhanced by adenosine triphosphate (ATP), indicating that its LLPS behavior can be fine‐tuned with proper strategy.^220^ Fusion transcription factors generated by genomic translocations have been recognized as important cancer drivers, especially for sarcomas and leukemias. FUS–CHOP, a FET oncofusion protein, undergoes PrLD‐mediated LLPS into liquid‐like condensates. It is well known that the N terminus of FUS is necessary for its LLPS, therefore, transcriptional activation by FUS–CHOP can account for the N terminus driving nuclear LLPS transition.

Furthermore, phase‐separated condensates of FUS–CHOP are colocalized with BRD4, a marker of super‐enhancer condensates,^221^ and FUS–CHOP recruits the chromatin remodeler SWI/SNF to forward global transcriptional reprogramming.^222^ It would be possible to target the LLPS and epigenetic remodeling complexes engagement capacities of FUS–CHOP confusion proteins to implement precision medicine for cancer therapy. EWSR1 also contains PrLDs to undergo LLPS, and O‐linked β‐D‐N‐acetylglucosaminylation (O‐GlcNAcylation) has been found to decrease the LLPS propensity of N‐terminal low complexity region of EWSR1.^223^ In addition, BRG1/BRM‐associated factor (BAF) with a PrLD has been found to interact with EWSR1. Following the LLPS transition, the BAF complex is recruited to tumor‐specific GGAA microsatellite repeat enhancers to activate target gene transcription.^216^ The formation of EWSR1 condensates can be intervened under different conditions. As for EWSR1–FLI1 takes the low complexity domain from EWSR1 and a DNA binding domain from FLI1. The low complexity domain of EWSR1–FLI1 is required for its undergoing LLPS and formation of protein granules in cells.^224^ EWSR1–FLI1 LLPS transition properties require retargeting the BAF chromatin remodeling complex and activating enhancers that drive the transcriptional program promoting cancer progression.^216^ This information sheds light on fine‐tuning the LLPS of FET fusion proteins with additional molecules.

### 5.2 Involvement of LLPS in heterochromatin formation in tumor cells

LLPS affects the formation of chromatin structure, which might ultimately contribute to the development of cancer.^225^ The chromatin of eukaryotes consists of positively charged histones and negatively charged DNA. Heterochromosomes are chromosomes in a cohesive state with tight morphology, gene deletions, high duplication of genomic regions, and transcriptional inertia.^226^ Heterochromosomes are primarily in the perisynaptic and telomeric regions, allowing for flexible gene silencing at multiple levels. Methylation of Histone H3 lysine 9 (H3K9me), a vital disability for posttranslational modification of histones, is a marker of heterochromatin formation. The heterochromatin protein family HP1 is an essential component of the dynamic nuclear response that senses and corrects defects in epigenetic information encoded in chromatin through histone modifications and DNA methylation. Defects in this crucial “chromatin repair” response in transformed human cells can be ideal targets for killing cancer cells. IDR‐containing HP1 and its homolog Swi6 can trigger LLPS on chromatin by recognizing H3K9me and forming heterochromatin.^227^ In contrast, the conformation of HP1 molecules altered by N‐terminal chromatin phosphorylation exposes the binding site, disrupts the molecular network structure, and inhibits LLPS.^228^ LLPS occurs based on multivalent interactions. Therefore, the forces and associated networks between multivalent molecules affect the structure and function of the related chromatin on the basis of LLPS.^229^, ^230^ Inhibition of HP1 LLPS by phosphorylation provides preferential elimination of cancer cells.^186^ In cancer cells, the nuclear morphology is frequently altered, a phenomenon known as atomic heterogeneity.^231^ Alternation in nucleoli's composition, number, size, and activity can provide a diagnostic basis for cancer. Because LLPS controls the integrity of part of the nuclear structure and the integrity of the microenvironment, modulating LLPS may play a key role in cancer therapy.

In eukaryotes, heterochromatin maintains genomic integrity and stability and is organized into distinct nuclear domains. DNA damage proteins can access heterochromatin by specific mechanisms, and erosion of heterochromatin by abnormal factors is linked to carcinogenesis. Tethering LLPS condensates with heterochromatin adds complexity to regulating the nuclear microenvironment. Heterochromatin binding protein HP1 has three homologs termed HP1α, HP1β, and HP1γ that function in DNA repair, nuclear organization, chromosome segregation, telomere maintenance, and gene silencing. HP1 might participate in many types of cancers and can serve as a potential biomarker for cancer prognosis and therapeutic target. Growing evidences have supported that HP1 proteins act as key modulators in micro‐LLPS and the segregation of heterochromatin‐like domains/complexes.^232^ HP1α and HP1β have been reported to form phase‐separated droplets to contribute to forming heterochromatin compartments.^233^, ^234^ Besides HP1, 53BP1 is another important player in regulating heterochromatin formation. It has been shown that 53BP1, together with HP1α undergoes LLPS at heterochromatin in a mutually dependent manner.^235^ Modulating the LLPS of factors, such as HP1 and 53BP1, to maintain heterochromatin integrity and genome stability can aid in preventing tumorigenesis and improve cancer therapy outcomes.

### 5.3 LLPS regulates gene expression to induce carcinogenesis and malignant behavior

Phase‐separated regulation of gene expression is involved in both transcription and translation processes. LLPS plays a critical role in both regulating DNA damage response pathway and RNA transcription process. LLPS regulates DNA‐responsive injury pathways to maintain genomic stability. DNA damage response and repair occur when signaling factors are activated and repair proteins are concentrated. 53BP1 is a scaffold for LLPS to bind DNA damage recognition and repair factor assembly to cell fate decisions.^184^ p53‐binding protein1 (53BP1) is a significant player in the DNA damage responding and repairing pathway and accumulates at DNA lesions, creating a nuclear environment called “53BP1 nuclear bodies” to scaffold factors for downstream signal cascades.^236^ p53 (a transcription factor) and USP28 (a deubiquitinase stabilizing p53) are assembled in 53BP1 by dynamic fusion and fission, symbolizing LLPS to encode an oncogenic factor.^237^ Generally, the 53BP1 nuclear body coordinates the DNA damage recognition with gene expression. Besides 53BP1, RAP80 LLPS has also been found to enhance the recruitment of BRCA1, a well‐known tumor suppressor gene, to DNA double‐strand breaks.^238^

Three different types of enzymes mainly regulate transcription, Pol I, Pol II, and Pol III.^239^ Pol I synthesizes preribosomal RNAs (rRNAs), Pol II produces messenger RNAs (mRNAs) and a variety of precursors for ncRNAs, and Pol III is responsible for transfer RNAs (tRNAs). LLPS differentially phosphorylates 239 RNA polymerase II at transcription initiation, elongation and termination to generate transcription factory or condensates.^194^ Upon entering the extension phase, RNA polymerase II dissociates from the transcriptional condensate in response to differential phosphorylation and relocates to another condensate used for splicing factors.^240^ These findings imply that LLPS is critical for transcription processes, including initiation and the switch to elongation, by concentrating factors and conferring dynamics with phosphorylation.^241^

In the first step of gene transcription, Pol II is a scaffold for the preinitiation complex, recruiting other factors to interact with itself and coordinate the transcription initiation. The CTD is a critical element of the scaffolding of Pol II and is highly structurally disordered, which is essential for the development of LLPS.^242^ CTD can promote LLPS through interactions with other regulators, and these binding sites enrich multiple transcriptional regulators in the condensate of CTD to achieve efficient activation of transcription initiation.^243^ CTDs can also be recruited to transcription centers formed by specific transcription factors to enhance transcription initiation.^192^, ^244^, ^245^ After escaping the promoter, Pol II is in a suspended state near the proximal region of the promoter.^246^, ^247^ To release from this proximal paused state, the cell cycle protein‐dependent kinase CDK9 phosphorylates residues of the Pol II CTD thereby removing the inhibitory effect of paused Pol II.^248^ CycT1 possesses an IDR and can fold into a structural domain through its N‐terminal region that binds tightly to CDK.^249^ CycT1 concentrates and compartmentalizes transcription factors into phase‐separated environments by LLPS through histidine‐rich structural domains within its IDR, leading to hyperphosphorylation of Pol II CTD and continued transcription elongation.^250^, ^251^ At the end of the regulatory transcription cycle, the Arabidopsis RNA‐binding protein FCA undergoes LLPS, in which the curly helix protein facilitates to reduce transcriptional read‐through.^252^, ^253^, ^254^ It can be concluded that LLPS participates in the whole process of gene transcription.

As a flexible and variable molecule, RNA is a potentially powerful provider of polyvalency in LLPS.^255^ lncRNA SLERT acts as an RNA modulator to regulate LLPS of fibrillar center/dense fibrillar component to facilitate Pol I transcription.^256^ Complex network structures formed by RNA–RNA interactions may directly trigger LLPS or provide a platform for protein coalescence and undergoing LLPS.^257^ Posttranscriptional modification of RNA regulates the properties and metabolism of RNA.^258^, ^259^ Numerous membraneless chambers promote gene regulation through various mechanisms, such as concentrating the activity of chelating factors to facilitate biochemical reactions or constructing interchromosomal hubs to promote the occurrence of macromolecules associated with gene expression.^31^, ^260^ Recently, several groups have reported that multivalent m^6^A‐modified RNAs act as scaffolds to gather YTHDF proteins and thus lead to LLPS both in vitro and in vivo.^261^ In addition, a membraneless organelle, the P‐body is involved in the regulation of translational repression and mRNA decay mechanisms. Changes in P‐body mediators affect mRNA metabolism and may lead to alternations in expression profiles and epigenetic regulation. P‐body mediators, such as miRNA and m^6^A, are associated with cancer.^18^ LLPS in RNA modification regulation would be a new research perspective because RNA biology is widely associated with various biological processes.

Abnormal transcriptional machinery is a widely accepted cancer hallmark. Therefore, based on the above description, it is easy to reach a consensus that LLPS holds a dominant role in cancer development. LLPS of RNA‐binding protein triggers the phosphorylation and release of Pol II to enhance active transcription.^262^ Many RNA‐binding proteins, such as TIA1, LIN28, ZEB1/2, RBM38, and TRBP, have been shown to be aberrantly expressed in cancers.^263^, ^264^ The transcriptional regulators’ YAP/TAZ can mediate cancer cells reprogramming into cancer stem cells, inciting carcinogenesis and cancer metastasis.^265^ LLPS of YAP fusions, YAP‐MAMLD1 and C11ORF95‐YAP have been considered the leading mechanism in initiating ependymoma from neural progenitor cells.^266^ Furthermore, inhibiting transcriptional coactivator activity mediated by condensates can prevent tumorigenesis,^266^ confirming the important role of YAP LLPS in oncogenic activity. SNHG9, a cancer‐promoting lncRNA, drives the formation of a liquid‐like droplet of LATS1 and downregulates the Hippo signaling.^267^ SNHG9 together with phosphatidic acids binds to LATS1 C‐terminal domain to trigger LAST1 LLPS, thus blocking LATS1‐mediated YAP phosphorylation.^267^ These results indicate a novel regulatory strategy to modulate cancer‐promoting transcription apparatus by facilitating LLPS.

In addition, the nuclear microenvironment of cancer is altered in response to LLPS.^268^ Super‐enhancers, which act as enhancer clusters to mediate dysregulation of transcriptional programs, are essential oncogenic drivers that sustain cancer cells.^269^, ^270^ Super‐enhancer‐rich transcriptional coactivators BRD4 and MED1 can form LLPS droplets at the super‐enhancer IDR, concentrating the factors in specific genomic regions.^270^ LSD1, also named KDM1A, is involved in the progression of multiple cancers. It has been demonstrated that LSD1 interacts with BRD4 and FOXA1 and is enriched at super‐enhancer regions, showing LLPS behavior.^271^ NUP98–HOXA9, a homeodomain‐containing transcription factor chimera in leukemias, contains IDR and can establish LLPS puncta of chimera.^200^ Phase‐separated NUP98–HOXA9 generates a “super‐enhancer”‐like binding pattern to potentiate transcriptional activation and induce leukemic transformation.^200^ NUP98–HOXA9 LLPS leads to the formation of proto‐oncogenes enriched CTCF‐independent chromatin loops.^200^ HOXB8 and FOSL1 are core regulatory circuitry components that control chromatin accessibility in super‐enhancers. HOXB8 and FOSL1 form dynamic phase‐separated droplets to incite the release of RNA Pol II from the promoter of super‐enhancer‐driven genes and the condensates can be specifically destroyed by GSK‐J4, a H3K27 demethylase inhibitor, to suppress metastasis and re‐establish sensitivity to chemotherapy treatment.^272^ LLPS remodels nuclear microenvironment provide mechanistic and therapeutic insights for targeting epigenetic factors to regulate chromatin accessibility in cancer therapy.

### 5.4 LLPS maintains cellular homeostasis to regulate cancer progression

The cell is separated from its surroundings by a cell membrane, and the internal conditions of the cell are very different from the fluid surrounding the cell. The cell constantly exchanges substances with the surrounding fluid and maintains its internal constancy, which is called cellular homeostasis. Cellular homeostasis includes proteostasis, redox homeostasis, calcium homeostasis, and so on. Cellular homeostasis has many essential roles in cancer therapy. For example, cellular metabolism can modulate the effector T cells to influence the adaptation of cellular functions in cancer immunity.^273^

**FIGURE 3:**
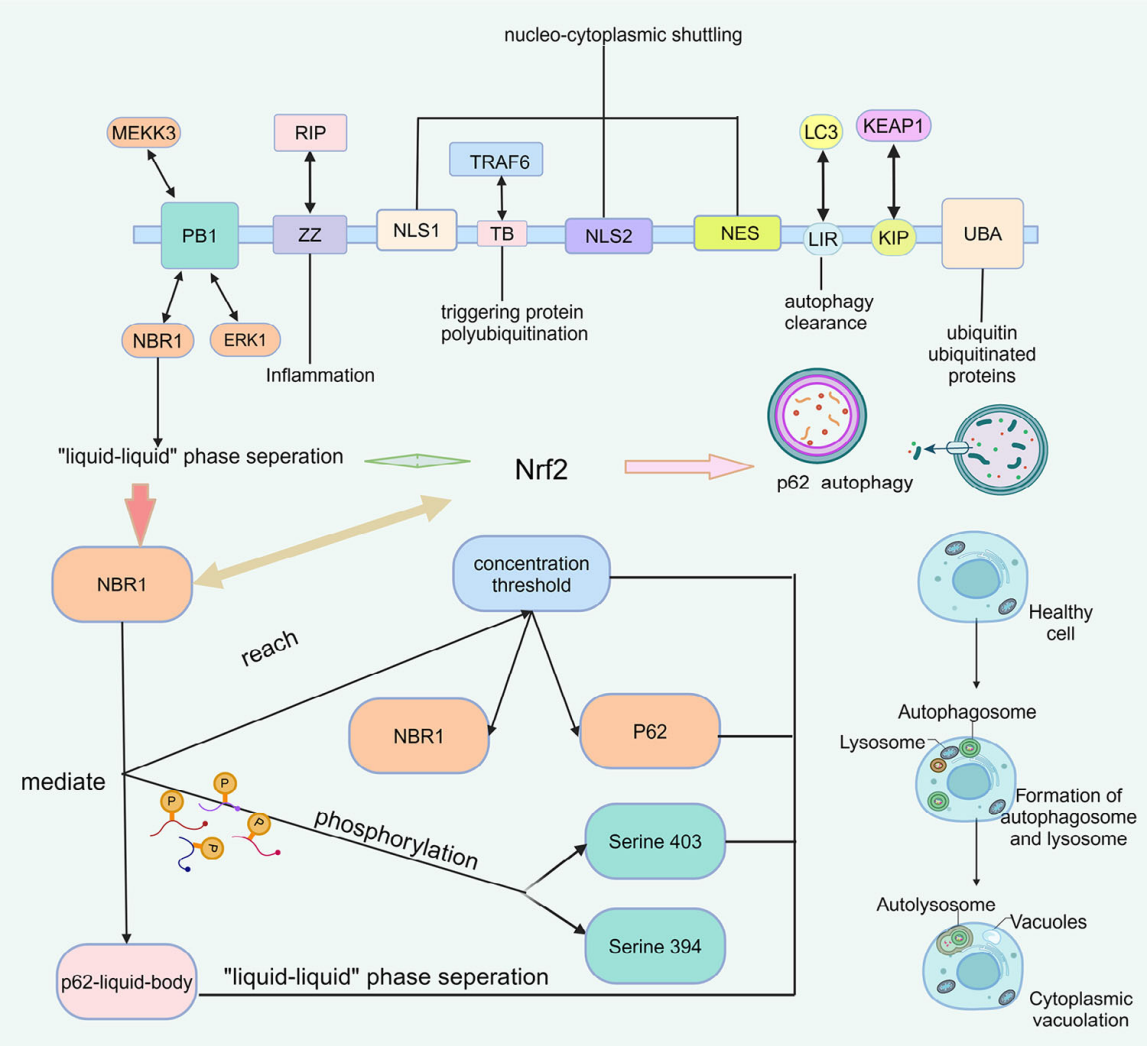
Structural composition and function of p62. p62 consists of an N‐terminal Phox‐BEM1 structural domain (PB1), a ZZ‐type zinc finger structural domain, a nuclear localization signal (NLS), an output motif (NES), an LC3 interaction region (LIR), a Keap1 interaction region (KIR), and a C‐terminal ubiquitin‐related structural domain (UBA). The boxes are interacting proteins, some of which undergo LLPS in response to p62, and posttranslational modifications of p62 regulate these functions. NBR1 promotes the formation of p62 liquid vesicles in cells. A possible mechanism is that the relative abundance of p62 and its binding partners exceeds a concentration threshold, and overexpression of NBR1 can increase the amount of p62 by activating Nrf62, leading to LLPS. Another possible mechanism is to improve the chemotaxis of p62 through posttranslational modifications. Posttranslational modifications, including phosphorylation, have been widely identified as regulators of LLPS.

Degradation of misfolded proteins is essential for cellular homeostasis. Selective autophagy mediates the degradation of harmful substances by separating these proteins into larger cohesions in stages through multivalent interactions and tethering them to the autophagosomal membrane.^274^ Membraneless particles(such as postsynaptic densities, signaling granules at DNA damage repair sites, and p62 granules involved in selective autophagy) have been found to assemble in membraneless compartments in several biochemical pathways and cellular pathways.^49^, ^275^, ^276^ During proliferation and spread, the intracellular environment of cancer cells, including the homeostasis of proteins, is altered. Gene activation or genomic instability impedes protein folding in cancer cells. Inadequate amino acid supply, glucose deprivation, and so on challenge protein processing in ER.^277^ p62 is a scaffolding and stress‐inducible protein. By regulating autophagy and apoptosis, this multifunctional protein controls cell viability in response to cytotoxic stress (Figure 3).^278^, ^279^, ^280^ Selective autophagy mediates the degradation of harmful substances by segregating these protein stages into larger endosomes through multivalent interactions and binding them to the autophagosome membrane. P62‐dependent hepatic differentiation is activated by autophagy inhibition. When the mTOR pathway is activated, p62 autophagy would be inhibited to cause protein accumulation. p62 is also closely associated with causing resistance to cancer therapy. Reduced p62 levels through autophagy upregulation in cisplatin‐resistant ovarian epithelial cancer have been observed to increase tumor sensitivity to the drug.^278^, ^281^

The oxidation and reduction reactions within the cell change the properties of the cellular components and provide energy to the organism. Redox imbalances in cells can lead to oxidative stress that impairs intracellular functions, for example, the susceptibility of the purine nucleotide guanine to oxidation, leading to DNA mutations that may initiate and propagate cancer.^282^ LLPS enables efficient energy production, promotes cell survival under acute hypoxia, and protects cells from apoptosis during cellular translational remodeling.^282^, ^283^ Nuclear factors erythroid 2‐related factor 2 (NRF2)‐mediated redox‐regulated pathways can sense cellular damage through LLPS and activate autophagy to degrade these damaged organelles. NRF2 has been shown to be a key activator of cancer‐supporting anabolism, and NRF2 activation has significant and protumor solid effects, particularly in the reprogramming of cancer cell metabolism.^284^

Calcium homeostasis controls autophagy in cancer cells. Ca^2+^ is a multifunctional second messenger, and as one of the most important factors regulating processes such as cell death, Ca^2+^ activates various regulatory proteins, including enzymes and transcription factors.^285^ Alterations in Ca^2+^ concentration in the cytoplasm and endoplasmic reticulum are integrated into the core through different regulatory mechanisms of autophagic activity.^286^ Ca^2+^ triggers the FIP200 complex to drive assembly via LLPS, triggering the formation of FIP200 sites, after which the autophagosome complex assembles in the endoplasmic reticulum.^287^ As a survival‐promoting and death‐inducing factor, autophagy boasts an excellent scope for tumorigenesis and prevention regulation.

### 5.5 LLPS remodels microenvironment to control cancer progression

**FIGURE 4:**
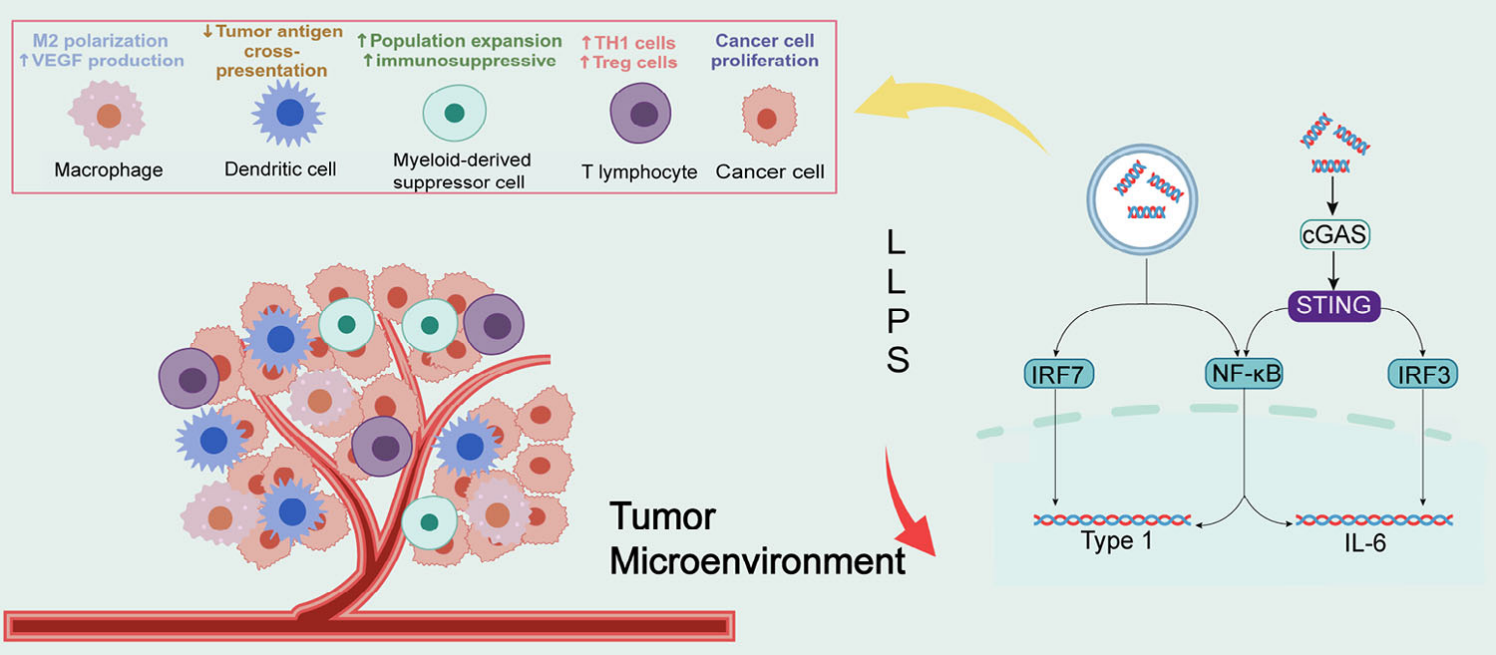
LLPS of the cGAS–STING pathway remodels tumor microenvironment. The complex tumor microenvironment consists of tumor cells, their surrounding immune and inflammatory cells, and other components. LLPS is involved in regulating intracellular immune signaling, especially the cGAS–STING pathway. The cGAS–STING pathway participates in tumor growth, angiogenesis, invasion, and metastasis.

The tumor microenvironment consists of tumor cells and their surrounding immune and inflammatory cells, tumor‐associated fibroblasts, nearby mesenchymal tissues, microvasculature, part of the neuronal regulatory circuits, as well as a variety of cytokines and chemokines, and is a complex integrated system.^288^, ^289^ In recent years, there is increasing evidence for the diversity of LLPS in the regulation of immune regulation, including the maturation and activation of immune cells, immune signaling, and so on.^47^ LLPS is also involved in the regulation of intracellular immune signaling, such as the cGAS–STING pathway (Figure 4).^290^ Cancer‐associated fibroblasts are essential components of the tumor microenvironment, and are activated to promote tumor growth, angiogenesis, invasion and metastasis. In addition, cancer‐associated fibroblasts can interact with tumor‐infiltrating immune cells and other immune components within the tumor microenvironment by secreting various factors and other effector molecules, resulting in immune effector cell dysfunction and suppression of antitumor immunity.^291^ Then, whether modulation of immune cells by LLPS and attenuation of their action with tumor‐promoting factors by immune components can inhibit cancer growth and invasion in the tumor microenvironment.

## 6 SYNERGY OF LLPS AND RNA MODIFICATIONS IN DISEASES

**FIGURE 5:**
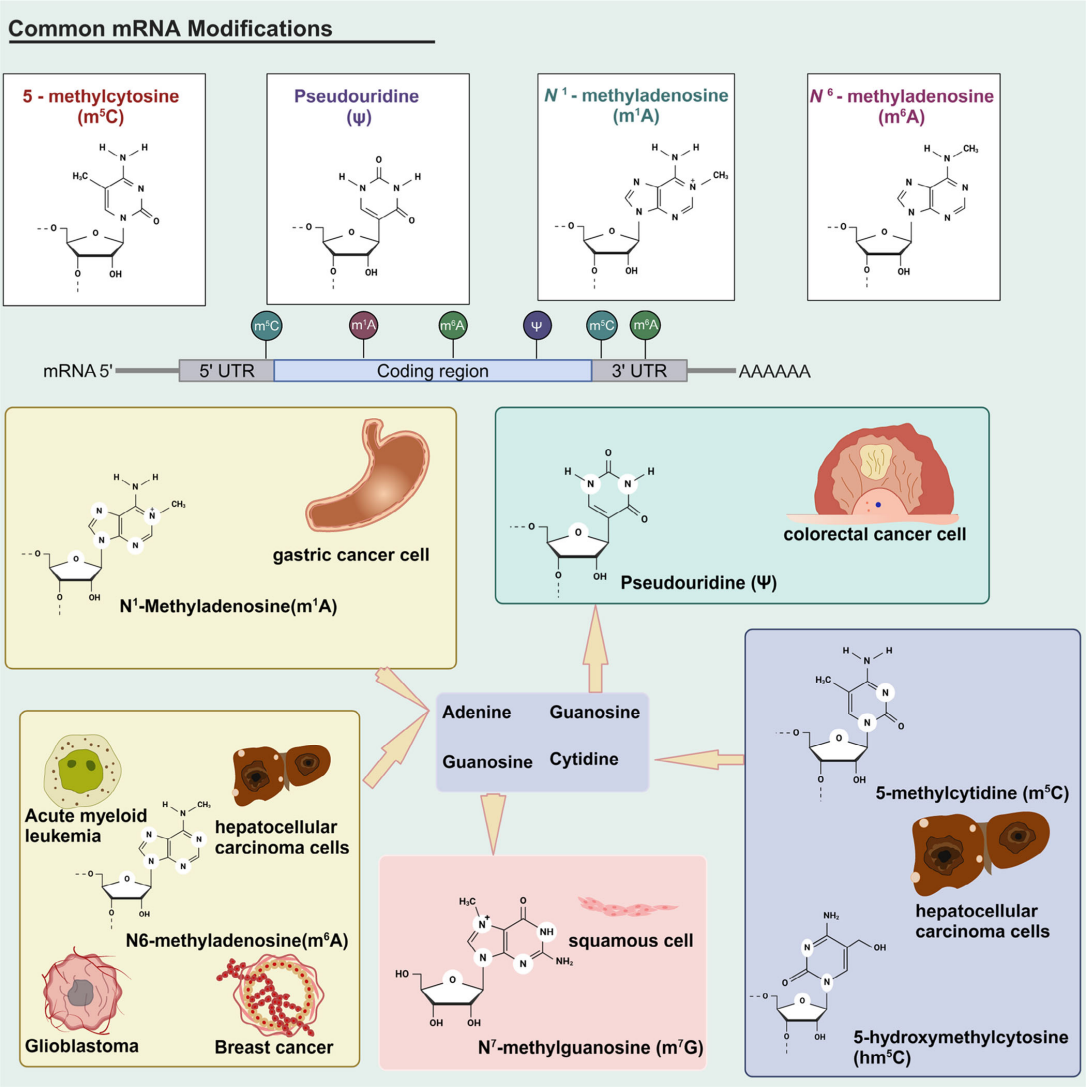
Chemical structure of RNA modifications and the major types of cancers primarily affected by these modifications. This figure summarizes the links in the main text between RNA modifications and various types of cancer. In addition, it also reflects the location where RNA modification occurs, and combining it with the chemical structure formula facilitates a more intuitive appreciation of the relationship between structure and function.

To date, more than 150 different types of chemical modifications of RNA have been identified. Common RNA modifications include m^6^A, m^1^A, 5‐methylcytidine (m^5^C), 5‐hydroxymethylcytosine (hm^5^C), adenosine‐to‐inosine editing (A‐to‐I), m^7^G, pseudouridine (Ψ), N^4^‐acetylcytidine (ac^4^C), and 2ʹ‐O‐methylation. Modifications of different RNA species, also named epi transcriptome, have emerged as a critical regulator of transcript expression, molecular function and homeostasis. All these RNA modifications might be involved in cancer pathogenesis (Figure 5). RNA modifications are involved in various signaling pathways, and several of these proteins play a role in regulating disease progression.^292^, ^293^, ^294^ RNA modifications (mainly m^6^A) have been reported for their protein LLPS,^255^ and they in turn, are strongly associated with pathological processes. Therefore, it is possible to fight against diseases by regulating LLPS of RNA‐modified related proteins.

### 6.1 N^6^‐methyladenosine

#### 6.1.1 Introduction of m^6^A

Discovered in the 1970s, m^6^A is the most abundant internal modification of mRNA and long‐stranded ncRNA (lncRNA) in most eukaryotes.^295^ Mostly, m^6^A modification occurs at the RRACH motif (R denotes A or G, H denotes A, C, or U). The formation of m^6^A is a reversible process involved in all aspects of RNA metabolism, including pre‐mRNA splicing, 3′‐end processing, nuclear export, translational regulation, mRNA decay, and ncRNA processing.^296^ Besides regulating the RNA cycle, m^6^A is widely involved in the metabolic reorganization of tumor cells and is an important influencing factor in many cancers. The principal function of m^6^A has been indicated to regulate the stability of targeted mRNAs. m^6^A‐associated proteins use different mechanisms to recognize and bind m^6^A. For example, YTH structural domain‐containing proteins (YTHDF1/2/3 and YTHDC1/2) use the YTH structural domain to recognize m^6^A, and LLPS regulates these proteins.^261^, ^297^ The effects of m^6^A are dynamically and reversibly determined by methyltransferases (writers), demethylases (erasers), and m^6^A binding proteins (readers).

*m^6^A writers*: Methyltransferase‐like 3 (METTL3), methyltransferase‐like 14 (METTL14) and Wilms’ tumor 1‐associated protein (WTAP) form the m^6^A methyltransferase complex, and their knockdown reduces m^6^A levels in polyadenylated RNA.^298^ Results of the experiment have shown that METTL3 and METTL14 form a stable METTL3–14 complex at a 1:1 stoichiometric ratio, and the dimer of the METTL3–14 complex induces m^6^A deposition on nuclear RNA. WTAP binds to the METTL3–14 complex, which has no methyltransferase activity but interacts with the METTL3–14 complex to influence m^6^A methyltransferase activity in vivo and location in nuclear spots.^299^

*m^6^A erasers*: Fat mass and obesity‐associated protein (FTO) and AlkB homolog 5 (ALKBH5) have been reported to exhibit demethylation activity.^300^ FTO and ALKBH5 belong to the α‐ketoglutarate‐dependent dioxygenase family and catalyze m^6^A demethylation in a Fe(ii) and α‐ketoglutarate dependent manner. Overexpressed FTO and ALKBH5 significantly reduce m^6^A levels in mRNA. ALKBH5 strictly selects substrates in the catalytic process, and the loss of ALKBH5 damages RNA metabolism, mRNA output, and assembly.^300^

*m^6^A readers*: To exert biological function, m^6^A needs to be identified by variable “readers” to exercise different downstream effects.^301^ Members of the YT521‐B homologous (YTH) domain family, including YTHDF1, YTHDF2, YTHDF3, YTH N^6^‐Methyladenosine RNA Binding Protein C1 (YTHDC1), and YTHDC2, recognize m^6^A and function as readers with certain members of the heterogeneous nuclear ribonucleoprotein (HNRNP) family. YTHDF1 promotes the translation of m^6^A‐methylated mRNA, YTHDF2 accelerates the decay of m^6^A‐methylated mRNA, and YTHDF3, together with YTHDF1 and YTHDF2, noticeably enhances the metabolism of m^6^A‐methylated mRNA in the cytoplasm.^302^ For HNR family, HNRNPA2B1 promotes primary miRNA processing and maturation by binding to m^6^A methylated transcripts, and HNRNPC and HNRNPG also regulate mRNA abundance and splicing by processing m^6^A modified RNA transcripts.^295^, ^303^, ^304^

#### 6.1.2 Dual role of m^6^A modification in cancer development and therapy

m^6^A methylation might act as a potent regulator of tumor metabolism (Table 3). In metabolic reprogramming in tumor cells, m^6^A methyltransferase and corresponding signaling pathways are essential modulators. m^6^A modification can play an active role in multiple pathways of tumor inhibition. However, for some signaling pathways, transcription factors, and enzymes associated with metabolic recombination, dysregulation of m^6^A methylation can damage cells or lead to treatment failure.^305^

**TABLE 3:** LLPS of m^6^A enzymes function in the progression of several types of cancers.

Type	Proteins	LLPS	Cancer	Mechanism (reference)
m^6^A Writers	METTL3	METTL3 phase separation regulates m^6^A methyltransferase complex assembly.^306^	Glioblastoma	High levels of METTL3 promote m^6^A transcription and increase ADAR1 protein levels, leading to cell cycle acceleration. Targeting ADAR1 in glioblastoma tumors completely inhibited tumor growth in vivo.^307^
			Glioblastoma	Overexpression of METTL3 significantly promoted the growth and invasion of bladder cancer cells.^308^
			Breast cancer	METTL3 promotes aberrant expression of mammalian hepatitis B X‐interacting proteins driving breast cancer aggressiveness.^309^
			Acute myeloid leukemia	METTL3 is a regulator of chromatin‐based pathways that is necessary for the maintenance of leukemic cells.^310^, ^311^
	METTL14	WTAP/WTAP‐ Vir like m^6^A methyltransferase associated (VIRMA)^312^ interacts with the METTL3–METTL14 condensate to influence methylation.^299^, ^313^	Hepatocellular carcinoma	METTL14 suppresses the metastatic potential of hepatocellular carcinoma by regulating m^6^A‐dependent primary microRNA processing.^314^
	WTAP		Acute myeloid leukemia	WTAP is important in the abnormal proliferation and differentiation arrest of leukemic cells.^315^
	VIRMA		Nasopharyngeal carcinoma	VIRMA enhances mRNA stability and transactivates integrin subunit alpha (ITGA2), promoting nasopharyngeal carcinoma progression.^316^
m^6^A Erasers	ALKBH5	The disordered C‐terminus of ALKBH5 promotes LLPS.^317^	Breast cancer	ALKBH5‐dependent approach induces increased NANOG mRNA and protein expression and breast cancer stem cell phenotype.^318^
			Glioblastoma	ALKBH5 is highly expressed in glioblastoma stem cell‐like cells.^319^
m^6^A readers	YTHDF2	YTHDF2 enhances the phase separation potential of mRNA.^261^	Acute myeloid leukemia	YTHDF2 inhibits the proliferation of stem cells into targeted mRNA decay.^320^
			Hepatocellular carcinoma	YTHDF2 inhibits the ERK/MAPK pathway to suppress cell proliferation.^321^
	YTHDF1	YTHDF1 promotes mRNA degradation via and LLPS.^297^	Ovarian cancer	YTHDF1 enhances EIF3C translation in an m^6^A‐dependent manner by binding to m^6^A‐modified EIF3C mRNA, thereby promoting tumorigenesis and metastasis in ovarian cancer.^322^
	YTHDF3	LLPS and proapoptotic effects of YTHDF3 can be modulated.^261^	Breast cancer	YTHDF3 promotes the interaction of breast cancer cells with brain endothelial cells and astrocytes, blood–brain barrier extravasation, angiogenesis, and growth.^323^
	YTHDC1	YTHDC 1 can undergo LLPS and form YTHDC1–m^6^A condensates.^324^	Bladder cancer	YTHDC1 regulates the positive expression of the PTEN/PI3K/AKT signaling pathway and enhances chemoresistance in bladder cancer.^325^

##### m^6^A modification inhibits tumor progression

Many studies have shown that downregulation of m^6^A promotes the progression of multiple tumors. Glioblastoma is the most‐deadly primary brain tumor. Glioblastoma stem cells can promote tumor growth and invasion, resist radiotherapy and chemotherapy, and drive a poor prognosis of glioblastoma. It has been found that decreased m^6^A levels in ADAM19 enhance its expression, promoting glioblastoma stem cell growth and self‐renewal and ultimately leading to tumorigenesis.^326^ Studies have shown that the progression of hepatocellular carcinoma is associated with abnormal m^6^A modification.^326^ METTL3 is frequently upregulated in hepatocellular carcinoma patients, and the upregulated METTL3 inhibits the expression of tumor suppressor SOCS2 through m^6^A methylation and promotes the development of hepatocellular carcinoma.^327^ METTL14‐mediated m^6^A modification of PTEN mRNA suppresses the progression of clear‐cell renal‐cell carcinoma.^328^ Pri‐miR126 is a direct substrate of METTL16. In hepatocellular carcinoma, the decreased m^6^A level on pri‐miR126 influences its maturation and disrupts the tumor suppressor function of miR126, accelerating tumor progression.^314^ In certain circumstances, restoration of METTL3 and METTL14 expression to re‐establish m^6^A distribution can inhibit cancer cell progression.

##### m^6^A modification promotes tumor progression

Some studies also reveal that m^6^A modification might exhibit tumor‐promotion properties. Acute myeloid leukemia is one of the most common hematological malignancies with apparent genetic aberrations and unsatisfactory therapeutic outcomes. METTL3 has been reported to play a carcinogenic role in acute myeloid leukemia. CEBPZ recruits METTL3 to the promoter region of SP1 to enhance the m^6^A modification of SP1 and stimulate its translation.^310^ SP1 then activates the oncogene c‐MYC, leading to the development of acute myeloid leukemia.^310^ Recently, METTL3 has been found to mediate the m^6^A modification of PTCH1 and GLI2 to control their RNA stability and translation to activate Sonic hedgehog signaling, resulting in the promoted progression of SHH subgroup medulloblastoma.^329^ METTL3‐mediated m^6^A modification decreases the expression of IFFO1, a novel tumor suppressor, via affecting mRNA stability to promote tumor development and cisplatin resistance in ovarian cancer.^330^ In addition to METTL3, METTL14 has also been shown to enhance tumorigenesis and be associated with poor prognosis in several types of cancers.^331^, ^332^ Knockdown of METTL14 inhibits the malignant progression in vitro and in vivo.^331^, ^333^ METTL3 and METTL14 may thus be a potential prognosis marker and a therapeutic target in certain types of cancers. Proper regulation of METTL3 and METTL14 may help restore m^6^A landmarks to halt cancer progression. Acetylation in METTL3 has been found to impede lung cancer metastasis by impaired m^6^A methylation.^334^

#### 6.1.3 m^6^A in other diseases

In addition to its role in cancer, m^6^A is also widely involved in diseases, including cardiovascular and neurological. Some research has found that mRNAs encoding protein kinases and many signaling molecules are hypermethylated in hypertrophied hearts.^335^ METTL3 has been reported to be involved in the regulation of epilepsy. Activation of METTL3‐mediated downregulation of Vim expression attenuates hippocampal neuronal damage and apoptosis and prevents epilepsy progression.^336^ Overexpression of METTL3 leads to hypermethylation of genes involved in cardiac hypertrophy.^337^ Also, m^6^A methylation plays an essential role in cardiac fibroblast differentiation, and METTL3 overexpression leads to enhanced differentiation of fibroblasts into myofibroblasts.^338^ Recently, the role of METTL14 has also been shown in vascular complications. METTL14 knockdown significantly reduces atherosclerotic plaque development.^339^ The expression of FTO and ALKBH5 is reduced in the failing heart and hypermethylation of m^6^A of these two genes regulates the translation of particular mRNA transcripts.^340^, ^341^ FTO gene expression is significantly reduced in both mouse and human failing hearts, and alterations in its expression may contribute to many cardiovascular abnormalities, including hypertrophic cardiomyopathy, arrhythmias, and coronary artery disease.^340^ Knockdown of ALKBH5, on the other hand, significantly reduced the expression of autophagy significant factors, which may be involved in autophagy and apoptosis in hypoxia‐treated cardiomyocytes.^342^ Besides writers and erasers, m^6^A readers also play a critical role in gene expression. Altered YTHDF1–3 expression may alter the translational efficiency of methylated transcripts.^302^ YTHDF2 inhibition significantly reduces inflammatory cytokines possibly involved in cardiovascular disease.^343^ m^6^A is increased in overall abundance in the brain and is involved in nerves related to embryonic brain development,^344^ learning and memory.^345^, ^346^ Deletion of METTL3 or METTL14 has a strong effect on glial cells, leading not only to prolonging the cell cycle of radial glial cells,^347^ but also a decrease in the number of glial cells.^348^ In the nervous system, FTO also has important effects with ALKBH5. FTO is highly expressed in adult neural stem cells and neurons and affects brain size, body weight, learning and memory.^346^ Under hypoxic conditions, the deletion of ALKBH5 disrupts many vital genes.^349^ YTHDF1 is increased in the hippocampus^350^ and promotes protein translation in response to neuronal stimulation.^351^

### 6.2 N^1^‐methyladenosine

m^1^A, another type of RNA methylation, is a crucial internal RNA modification that controls gene expression, appearing on the first nitrogen atom of adenosine in RNA.^352^ Similar to m^6^A, m^1^A can be installed by “writer” methyltransferases, removed by “eraser” demethylases and recognized by m^1^A‐binding proteins called “reader.”^353^ m^1^A is very abundant in tRNAs and is most conserved and common in bacteria, archaea and eukaryotes.^354^ m^1^A at position 58 is the first m^1^A modification of the initial transcripts of tRNA in the nucleus, whose methyl methyltransferase complex has been first discovered in Saccharomyces cerevisiae.^355^ m^1^A plays an important role in the regulation of pre‐RNA processing, RNA structure, translation and stability.^356^ It should be noted that the biological function and modification sites of m1A modification vary in different species.

m^1^A is involved in regulating disease initiation and progression. m^1^A methylation upregulates microfibril‐associated protein 2 (MFAP2) expression and promotes colorectal cancer metastasis by blocking autophagic degradation of CDC Like Kinase 3 (CLK3).^356^ In gastric cancer cells, m^1^A regulates the PI3K/AKT/mTOR pathway and ErbB pathway. Knockdown of ALKBH3, the “writer” protein of m^1^A, has been observed to result in poor prognosis and metastasis in gastric cancer.^357^ In addition, m^1^A is also involved in the prognosis of gliomas.^358^ m^1^A‐related proteins, including ALKBH1, TRMT6, TRMT10C, and YTHDF1, are significantly overexpressed in gliomas. TRMT6 regulates cell cycle distribution and increases glioma cell proliferation and death.^358^, ^359^ m^1^A methylation in mitochondria and cytoplasm has been reported to be dysregulated in AD.^360^, ^361^ For example, aberrant m^1^A deposits lead to complex I dysfunction in AD.^362^ In sum, there has been increasing evidence of alterations in m^1^A occurring in various diseases, and these m^1^A modifications may be potential drug targets.

### 6.3 m^5^C and hm^5^C

In eukaryotes’ RNA, m^5^C and hm^5^C are the prominent modifications and are thought to contribute to epigenetic regulation through various mechanisms.^363^ NOL1/NOP2/NSUN family and DNMT2 catalyze m^5^C, which covalently transfers methyl from S' adenosine methionine to the cytosine pyrimidine ring.^363^, ^364^ m^5^C methylation is a reversible epigenetic modification with a constant turnover of cytosine modifications.^365^ This dynamic regulation of reversible chemotaxis is important in cell fate determination and can act as an epigenetic barrier by limiting the developmental potential of cells and restricting their differentiation.^366^

The methyltransferase of m^5^C has been observed in a variety of cancers, including glioma, gastric cancer and leukemia.^364^ In hepatocellular carcinoma cells, the methyltransferase NSUN2 of m^5^C can target the tumor‐associated gene lncRNA H19 to promote tumorigenesis and progression. G3BP1 regulates a variety of tumorigenesis‐related pathways, including Ras, Wnt/β‐catenin, PI3K/AKT, and NF‐κB/Her2 signaling pathways. RNA purification and mass spectrometry analyses indicate that m5C modifications regulate the binding of G3BP1 protein to H19 RNA.^367^ In addition, NSUN2 is upregulated in gastric cancer and promotes cancer cell proliferation, migration and invasion. SUMOylation is a significant regulatory posttranslational modification, and its covalent bond interaction with NSUN2 to form the SUMOylation–NSUN2–m^5^C axis is a novel mechanism and therapeutic target for gastric cancer cells.^368^ The methyltransferase DNMT2, in turn, can interact with RNA‐binding proteins to mediate the 5‐azacytidine response in leukemia.^369^ Azetidine acrylamides, stereoselective covalent inhibitors of human NSUN2, can cause a global reduction in tRNA m^5^C content in cancer cells.^370^ Growing studies are currently undergoing to unveil the role of m^5^C modification in cancer development.

NSUN2 are all enriched in the developing brain^371^ and are knocked down to delay or block late differentiation in specific tissues including skin and testis.^372^ Several mutations in NSUN2 may cause growth and mental retardation, unusual facial and skin abnormalities.^373^ And mutations in NSUN3 have been reported to be detected in patients with mitochondrial‐deficiency disorders.^374^, ^375^ NSUN5 is located in the deletion genome of a neurodevelopmental disorder called Williams‐Beuren syndrome,^376^ and the deletion of NSUN5 may be one of the pathogenic causes of the disorder.^377^ NSUN7 is widely expressed in testicular cells and may lead to reduced sperm viability under induced mutations.^378^ In summary, m^5^C is commonly involved in a variety of diseases.

As the hydroxylated form of m^5^C, hm^5^C is now widely recognized as the “sixth base” of the mammalian genome.^379^, ^380^ The hm^5^C modification has also been identified in RNA, and ten‐eleven translocation (TET) enzymes are responsible for the oxidation of m^5^C into hm^5^C in RNA.^381^, ^382^ It has been found that the deposition of hm^5^C on tRNA depends on TET2, and TET2 knockout impairs the abundance of several ncRNAs.^383^ In a recent study, it has been confirmed that ALKBH1 is the primary enzyme for hm^5^C modification in RNA other than TET2.^384^ It is well known that both TET2 and ALKBH1 are essential for cancer initiation and progression. Besides hm^5^C, there are also different types of oxidative metabolite of m^5^C in RNA, including 5‐Formylcytidine (f^5^C)^385^ and 2′‐O‐methyl‐5‐hydroxymethylcytidine (hm^5^Cm).^384^, ^386^ However, the biological function of these modifications in RNA remains largely unknown.

### 6.4 Adenosine‐to‐inosine editing

Irreversible deamination reaction A‐to‐I converts adenosine to inosine in the dsRNA region, thus functionally acting as an A‐to‐G mutation.^387^, ^388^ Adenosine deaminase acting on the RNA (ADAR) family is the most prominent A‐to‐I modifying enzyme and is dysregulated in many human diseases.^389^

ADAR1, which is much more enriched than ADAR2, plays the most important role in the family and is closely associated with cancer development.^390^ ADAR1 recognizes the hairpin structure of dsDNA and edits it, leading to inactivation or activation of the final translation product and disrupting miRNA binding sites to alter mRNA stability.^391^ Knockdown of ADAR1 results in mice exhibiting massive apoptosis, defective erythropoiesis, and abnormal innate immune responses.^392^ ADAR2 enzyme targets the Q/R locus in the mammalian central nervous system in the mouse brain, and defects at this locus may result in cell death of excitotoxic neurons, leading to excitotoxicity.^393^, ^394^ As an important target of ADAR1, Antizyme inhibitor 1 (AZIN1) is involved in forming various cancers, including colorectal and lung cancer. Takeda et al.^395^ proposed a new mechanism by which AZIN1 promotes the accumulation of proteins necessary for polyamine synthesis, activating and increasing fibroblast invasion into the tumor microenvironment. AZIN1 promotes tumor angiogenesis in colorectal cancer by upregulating IL‐8, and the IL‐8 receptor profoundly affects the tumor microenvironment, increasing the proliferation, survival, and migration of vascular endothelial cells and the permeability of endothelial cells.^396^

### 6.5 N^7^‐methylguanosine

m^7^G is the RNA methylation of guanine at the N7 position, which primarily occurs in the cap region of mRNAs with RNAs and tRNA internals.^397^ The METTL1/WDR4 complex appears to be the “writer” in m^7^G modification.^398^ Although METTL1 is mainly responsible for mediating m^7^G methylation, WDR4 has more favorable binding sites on RNA for stabilization.^399^ In cancer, METTL1 is frequently overexpressed in inducing oncogenic cell transformation. Arg‐TCT‐4‐1, the m^7^G‐modified tRNAs, can increase translation of AGA codon‐rich mRNAs and control tumorigenesis and growth, and the upregulated phenotype of tRNA–Arg–TCT‐4‐1 has been shown to be a METTL1/WDR4 overexpression phenotype.^400^, ^401^ A missense mutation in WDR4 may be the causative variant in microcephalic primordial dwarfism, a neurological disorder with facial deformities and brain malformations.^394^ Furthermore, WDR4 expression levels were significantly reduced in hippocampal tissues of Down syndrome mice,^402^ and overexpression of WDR4 promoted cognitive function and hippocampal plasticity.^403^ Mutations in METTL1/WDR4 cause neurodevelopmental disorders through multiple mechanisms.^404^ Furthermore, in head and neck squamous cell carcinoma, METTL1 accelerates cancer progression by promoting the translation of cell cycle proteins and related oncogenes through the PI3K/AKT/mTOR signaling pathway.^405^ The METTL1/WDR4 complex continues to promote oncogene translation and regulates the progression of cell cycle‐related proteins in lung cancer through a codon‐dependent manner.^406^ Hence, targeting m^7^G to inhibit oncogene translation would be a potential antitumor strategy. WBSCR22/TRMT112 is another significant m^7^G methyltransferase complex.^407^, ^408^ As an m^7^G writer, WBSCR22 has been reported to be involved in organ regeneration and enhancing glucocorticoid receptor function in lung cancer.^404^, ^409^, ^410^ In addition, WBSCR22 has been shown to affect tumor cell survival and drug resistance in a variety of cancers, including breast cancer, melanoma, and colorectal cancer.^411^, ^412^, ^413^, ^414^ TRMT112 is a cofactor of WBSCR22, maintains its stability and aids in m^7^G modification installation.^407^, ^415^ The information concludes that m^7^G modifications and related methyltransferases function in regulating disease development and drug resistance.

### 6.6 Pseudouridine

The C5‐glycoside isoform of uridine, Ψ, deposits in both coding and noncoding regions of RNA and is highly conserved among species.^416^ Ψ in tRNA contributes to its structural stabilization with inhibition of protein synthesis.^417^, ^418^ Possibly, since C‐C is more inert than C‐N, Ψ’s “reader” and “writer” proteins are less detectable than other modifications.∖Mutations in the DKC1 enzyme in the Ψ‐mediated catalytic mode by RNA‐dependent mechanisms are associated with aberrant protein translation and have been reported to be upregulated in a variety of cancers.^407^, ^419^ Impaired telomerase activity and gene expression in the absence of DKC1 activity leads to decreased proliferation and survival of cancer cells.^420^ DKC1 is significantly upregulated in colorectal cancer tissues, acting as a critical regulator for cancer cell proliferation. Three hundred eighty‐three thousand three hundred eighty‐four rRNA mutants are very common in colorectal cancer,^421^ so ψ modification of reduced ribosomes may affect translation in cancer cells. Ψ can regulate the misreading of mRNA, and might have a unique application in the development of RNA drugs.

### 6.7 N^4^‐acetylcysteine

ac^4^C is currently the only type of acetylation modification in eukaryotic RNAs, mainly occurring on tRNAs and rRNAs and, to a lesser extent, mRNAs.^422^ ac^4^C in tRNA helps to maintain translational stability and plays a role in thermophilic bacteria for their heat resistance.^423^, ^424^ ac^4^C is vital in the diagnosis and treatment of cancer. Studies have found that cancer patients have a more pronounced increase in ac^4^C than standard nucleosides and that ac^4^C levels are significantly lower after tumor resection.^425^ In addition to cancer diagnostics, ac^4^C has a regulatory role in bladder, gastric, and colorectal cancers.^426^, ^427^ N‐acetyltransferase 10 (NAT10) exhibits acetyltransferase and RNA binding activity and can catalyze ac^4^C modification. NAT10‐mediated acetylation improves mRNA stability and translational efficiency.^428^ High expression of NAT10 is necessary for the tumorigenic properties of bladder cancer. Regulation of ac^4^C via NAT10 involves proliferation, migration, and stem cell properties of bladder cancer cells.^429^, ^430^ In gastric cancer, ac^4^C acts as the inducer of hypoxia tolerance through glycolytic mechanisms. NAT10 receives regulation of a critical transcription factor HIF‐1α in the hypoxia response, which triggers the activation of the HIF‐1 pathway and regulates ac^4^C to enhance hypoxia tolerance in gastric cancer cells.^431^ NAT10 mediates an increase in ac^4^C modification and promotes the binding of polysaccharides, which enhances translational efficiency and induces central sensitization for neuropathic pain.^432^ Knockdown of NAT10 significantly reduced macrophage inflammatory responses, whereas overexpression resulted in the opposite result. It has been demonstrated that NAT10 regulates macrophage inflammatory responses through the NOX2–ROS–NF‐κB pathway.^433^ The abundance of ac^4^C modifications in lncRNA significantly differed in AD as obtained by highly repetitive sequence analysis.^434^ These studies highlight the critical role of ac^4^C in cancer development by controlling various pathological events, such as glucose metabolism reprogramming and cancer hypoxia.

### 6.8 2ʹ‐O‐methylation

2ʹ‐O‐methylation is the main methylation of the 2′‐OH portion of the ribose and is widely present in tRNA, rRNA and mRNA in mammalian cells.^435^ Absence of 2′‐O‐methylation in vertebrate development leads to severe morphological defects and embryonic lethality.^422^, ^436^ Fibrillarin is a 2′‐O‐RNA methyltransferase located in the dense proto‐fiber component of the nucleolus accumbens.^437^ Fibrillarin is an essential nucleolin protein involved in pre‐rRNA processing and regulates rRNA transcription, and it is overexpressed in different cancers.^438^ 2ʹ‐O‐methylated modification in ncRNAs is involved in colorectal carcinogenesis.^439^ Among these ncRNAs, SNORD11B functions in cancer proliferation and invasion and increases the processing and maturation of 18S rRNA by introducing 2ʹ‐O‐methylation on 18S rRNA G509 site. lncRNA ZFAS1 is significantly overexpressed in several human malignant tumors, including colorectal, hepatocellular, and gastric cancers, and knockdown of ZFAS1 inhibits cancer cell proliferation and migration and increases apoptosis.^440^ Overexpression of NOP58 reverses this inhibition of ZFAS1 and promotes 2ʹ‐O‐methylation.^441^ Another small nucleolar RNA, SNORD104, can be knocked down by antisense oligonucleotide in Ishikawa cells to reduce proliferation and migration in endometrial cancer.^442^ In this study, SNORD104 is found to bind to the 2′‐O‐methyltransferase fibrillarin and increases PARP1 2′‐O‐methylation.^442^ In malignant melanoma and acute myeloid leukemia, 2ʹ‐O‐methylation has also been recognized as a new target for treatment.^443^, ^444^ Loss of function of FTSJ1, a characteristic 2′‐O‐methyltransferase targeting tRNA, has been identified as the cause of nonsyndromic X‐linked intellectual disability.^445^, ^446^ Another 2′‐O‐methyluridine methyltransferase, TRMT44, is associated with idiopathic epilepsy.^447^ It still demands more studies to understand the role of 2ʹ‐O‐methylation in preventing and treating diseases.

### 6.9 LLPS in RNA modifying enzymes

Many RNA modifications have their own “reader,” “writer,” and “eraser” proteins that play an important role in the relationship between previously mentioned RNA modifications and diseases. For example, YTHDC1 undergoes LLPS requiring m^6^A, and the nuclear YTHDC1–m^6^A condensates maintain cell survival and the undifferentiated state of acute myeloid leukemia cells.^324^ Possibly, m^6^A acts as a bridge between LLPS and RNA to fine‐tune the subcellular localization of RNA to implement or even modify its functions in controlling cancer cells' occurrence, proliferation, apoptosis, and metastasis.^178^ Proteins with disordered structure are likely to undergo LLPS or have some involvement in forming membraneless organelles. As the m^1^A “writer” protein TRMT6/61A is significantly enriched in SG‐chelated mRNAs, it was shown that TRMT6/61A methyltransferase localizes to SGs under stress and protects mRNAs during heat shock.^448^ ALKBH5 specifically binds to proteins in the paraspeckles and forms phase‐separated droplets that bind to the paraspeckles through its C‐terminal IDR.^317^ In a hypoxic environment, proteins in the paraspeckles before the upregulation of ALKBH5 expression are induced with its rapid condensation to promote the formation of paraspeckles, reflecting an essential role in hypoxia induction.^448^ ADAR1 is localized to SGs formed in response to arsenite‐induced oxidative stress or long dsRNA transfection.^449^ METTL3 has a large number of disordered structures that are not only capable of LLPs independently but also of stabilizing METTL14 to bind constitutively to each other and form heterodimers. In addition, certain LLPS behaviors of METTL3 cancer mutants have now been observed.^306^ DNMT2 can relocate from the nucleus to SGs and participate in the protein processing of RNA.^450^ In sum, many RNA‐modifying enzymes harbor IDRs that must be explored in their association with LLPS.

### 6.10 Relationship between RNA modifications and LLPS

The phase boundaries of condensates can be controlled by changing RNA length, sequence, structure, modifications, and RNA–RNA and RNA–protein interactions.^255^ The use of RNA–RNA and RNA–protein interactions to influence RNA‐driven LLPS realizes that it is possible to use RNA to control condensates, such as the LLPS regulation of m^6^A.^451^ The RNA modifications can alter LLPS behaviors by refining RNA–RNA and RNA–protein interactions.

As mentioned earlier, membrane‐free fluidic regions play a role in many cellular functions; they are often enriched in RNA and influence the transcriptome. For example, nuclear speckles, which are significant regulators of gene expression and contain proteins and RNAs involved in gene expression, play a role in some cancers, and speckles, working together with p53, can directly enhance gene activity. Speckle‐associated p53 target genes are more likely to be involved in tumor suppressor functions, such as stopping cell growth and triggering cell suicide, than other p53 target genes.^452^, ^453^ Among them, nuclear speckles or splicing speckles, also called interchromatin granule clusters, are meaningful RNA‐containing membraneless chambers that function as sites for RNA splicing and RNA m^6^A methylation. LLPS of these compartments selects biomolecules to become concentrated, presumably to implement their functions.^454^, ^455^ Phase‐separated multivalent interactions are mediated by scaffold proteins with tandem repeat binding sites with other partners or proteins with IDRs (or low‐complexity structural domains), often in conjunction with nucleic acids.^253^ The m^6^A can be recognized by the YTH structural domain for LLPS in the IDR, and the LLPS potential can be further facilitated by multiple m^6^A modifications to the RNA.^261^

The YTH family of binding proteins of m^6^A consists of a m^6^A‐binding YTH domain of approximately 15 kD and a low complexity domain of approximately 40 kD.^298^ Under the action of LLPS, some low‐complexity structures (e.g., YTHDF family) form protofibrils, hydrogels or droplets.^149^ The heated sample of YTHDF proteins becomes turbid when warmed to 37°C, indicating that the warming causes lower critical solution temperature LLPS. Adding as low as 10% glycerol and reducing the salt concentration decreases the YTHDF2 concentration required for the LLPS transition to 1−8 µM.^261^ These results demonstrate that YTHDF2 LLPS is enhanced by increasing protein concentration but weakened by salt. Furthermore, studies reveal that polymethylated m^6^A‐RNA provides a scaffold to juxtapose multiple YTHDF proteins, causing these proteins to undergo LLPS through interactions between their low‐complexity domains.^261^ It possibly demonstrates that m^6^A‐RNA binding to the YTH domain regulates LLPS.^261^

YTHDF proteins localize to neuronal RNA granules, P‐bodies, and SGs, each of which is considered to be a phase‐separated compartment in the cytosol raising the possibility that LLPS may govern the localization of these proteins and potentially m^6^A‐mRNA. It is well known that nearly all m^6^A formation in mRNA is catalyzed by the METTL3–METTL14 heterodimeric methyltransferase.^456^ METTL14 knockout has been conducted to examine the effect of m^6^A‐mRNA on YTHDF2 protein localization to SGs and P‐bodies. Results reflected that METTL14 knockout did not alter the formation of SGs, but there was less YTHDF2 relocalization in SGs.^261^ In addition, the YTHDF2 mutant that lacks m^6^A binding exhibited less relocalization to SGs in wild‐type cells, indicating that binding to m^6^A mRNA is necessary for YTHDF2 to partition into SGs efficiently. YTHDF2 does not enrich the P‐body in the cells with METTL14 knockdown, suggesting that YTHDF2 being included in P‐bodies to form complexes is accompanied by m^6^A‐mRNA. In SGs, m^6^A‐mRNA occupies a higher percentage than in total cellular mRNA, further confirming that polymethylated m^6^A RNAs play an important role in LLPS.

Additionally, it is found that, unlike other forms of RNA‐scaffolded LLPS, m^6^A regulates the fate of cytoplasmic m^6^A through the scaffold YTHDF protein, leading to the formation of phase‐separated YTHDF‐m^6^A‐mRNA complexes that then partition into phase‐separated structures in cells.^261^ This series of experiments by Rise et al. demonstrated that m^6^A regulates the fate of mRNA by scaffolding YTHDF proteins to form phase‐separated YTHDF–m^6^A–mRNA complexes. The presence of m^6^A enhances RNA–RNA and RNA–protein interactions, targeting different intracellular condensates. Furthermore, O‐GlcNAcylation modification can occur on YTHDF1 and YTHDF3 to modulate the stability, assembly and disassembly of SGs.^457^ The regulatory mechanism of O‐GlcNAcylation in RNA m^6^A methylation highlights additional complexity to the posttranscriptional regulation function of LLPS.

Investigation shows that the LLPS of YTHDC1 and 3′ end processing factor FIP1L1 can be enhanced by binding to the m^6^A sites, probably playing a crucial role in their interaction and APA regulation.^458^ LLPS of YTHDC1 also undergoes liquid‐like condensates in highly active enhancers marked by m^6^A‐enhancer RNAs.^451^ YTHDC1/m^6^A‐enhancer RNAs condensates can further facilitate BRD4 coactivator condensates formation to implement enhancer activation and gene transcriptional control.^451^ Considering these essential findings, m^6^A might provide a regulatory LLPS mechanism based on m^6^A multivalency, potentially a new strategy for cancer treatment.

RNA is a crucial component of phase‐separated cellular compartments, and large amounts of protein‐free mRNA can be released from multimers into the cytoplasm under stress conditions.^459^ Free RNA is susceptible to aberrant interactions with misfolded proteins and undergoes protein accumulation, inducing the formation of irreversible RNA–protein coaggregation. Participation of m^1^A in mRNA granulation and protection during stress via TRMT6/61A methyltransferase.^14^ Strong binding of TDP‐43 to m1A stimulates cytoplasmic mislocalization and formation of TDP‐43 gel‐like aggregates.^460^ TDP43 promotes stemness in breast cancer stem cells, and deletion of TDP43 inhibits the progression of triple‐negative breast cancer.^461^, ^462^ m^7^G is predominantly present in mRNA processing and protein synthesis. Within mRNAs, m^7^G can be selectively recognized by quiver proteins (QKIs). m^7^G regulates mRNA stability and translation under stress conditions, and the bound complex QKI7 interacts with SGs core proteins (via the C‐terminus) and shuttles internally. Through extensive experimental validation, Zhao et al.^15^ found that QKIs can regulate target mRNA metabolism and cellular drug resistance by binding m^7^G. Also, A‐to‐I editing has been shown to be associated with alterations in the interaction sites of proteins involved in LLPS.^16^ However, there are still plenty of unknowns in investigating the relationship between RNA modifications and LLPS. The phenomenon and underlying mechanism of many RNA modifications in LLPS have not yet been discovered.

### 6.11 RNA modifications regulate LLPS to affect disease development

As mentioned above, m^6^A enhances the LLPS potential of mRNA, which in turn regulates various metabolic processes in organisms.^261^ Considering that both LLPS and m^6^A might participate in multiple pathological progression, it would be reasonable to hypothesize that LLPS can be regulated by m^6^A to control disease development.

Human papillomavirus (HPV), a diverse family of small double‐stranded DNA viruses, is the major risk factor for various types of cancers, including cervical cancer, head and neck cancer, and nonmelanoma skin cancer. The E7 protein encoded by high‐risk HPVs contributes to HPV‐associated carcinogenesis through multiple signaling pathways. Viral E7 mRNA contains modified m^6^A that IGF2BP1 stabilizes in HPV‐infected cells.^463^ Heat treatment has been reported to reverse HPV‐associated tumorigenesis both in vitro and in vivo, which enhances IGF2BP1 aggregation depending on the presence of m^6^A‐modified E7 mRNA to generate obvious m^6^A E7 mRNA–IGF2BP1 granules,^463^ indicating that controlling LLPS is a possible cancer therapeutic strategy in the m^6^A context. RNA driving IGF2BP1 LLPS has also been documented in several research studies.^464^, ^465^ IGF2BP1 is an important m^6^A modulatory gene that has always increased in many malignant tissues. RBM15 contains IDR and potentially forms “liquid‐like” condensates in vivo and in vitro.^466^ RBM15 condensates are prone to contact or partially overlap with nuclear speckles rather than other membraneless chambers.^466^ Most importantly, RBM15 condensates promote the m^6^A modification of the transcripts of highly expressing cancer‐related genes and are partially colocalized with m^6^A‐modified transcripts in the nucleus.^466^ In myeloid leukemia, m^6^A is found to be required for YTHDC1 to undergo LLPS, generating nuclear YTHDC1–m^6^A condensates to maintain cell survival and myeloid leukemic undifferentiated state.^324^ METTL3 can also undergo LLPS, and its self‐interacting ability provides the necessary multivalency for LLPS of mRNA m^6^A methyltransferase complex.^306^ Researchers also investigated the LLPS behavior of METTL3 cancer mutants, including R508H, R415C, and E516K, finding that R415C and E516K mutants fail to undergo LLPS separation.^306^ Because the METTL3 has been documented to function in many types of cancers,^329^, ^334^ modulating its LLPS behavior may be a potentially critical facet for inhibiting cancer progression.

**FIGURE 6:**
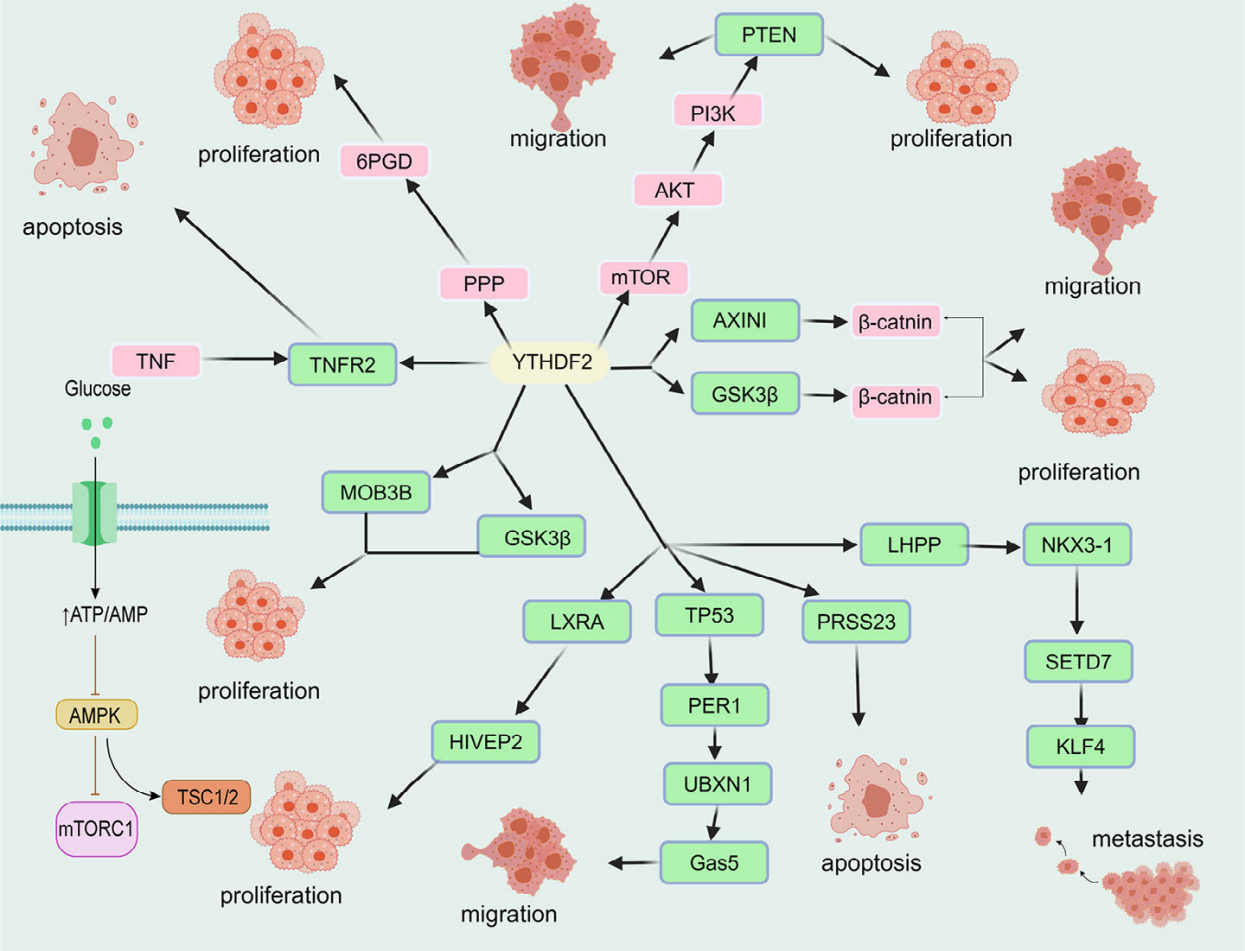
Signaling cascades underlying YTHDF2 in tumor proliferation, invasion, migration and metastasis. These signaling pathways include pentose phosphate signaling, PI3K/AKT, Wnt/β‐catenin, miRNA modulating pathway, and other m^6^A‐dependent pathways. The role of YTHDF2 is associated with the inhibition of tumor necrosis factor (TNF), which suppresses apoptosis in tumor cells. Knockdown of YTHDF2 results in the upregulation of TNF receptor 2 (TNFR2). The pentose phosphate pathway (PPP) plays an important role in regulating tumor cell growth by providing cells with ribose 5‐phosphate and NADPH through its rate‐limiting enzyme 6‐phosphogluconate dehydrogenase (6PGD); activated phosphoinositide‐3‐kinase (PI3K) triggers AKT activation, which activates mammalian target of rapamycin (mTOR); glycogen synthase kinase 3β (GSK3β) is an essential component of the Wnt/β‐catenin pathway, and its inactivation leads to the intracellular accumulation and translocation of β‐catenin to the nucleus of the cell, thereby promoting tumor progression. In addition, AXIN1, which encodes a negative regulator of the Wnt/β‐catenin pathway, has been identified as a direct target of YTHDF2; YTHDF2 modulates the expression of tumor suppressors in an m^6^A‐dependent manner; and miRNAs modulate YTHDF2 expression.

Many cancer‐related genes can undergo LLPS and be regulated by m^6^A modification. Low‐complex domain RNA‐binding protein FUS can mediate LLPS.^467^ FUS is overexpressed in prostate adenocarcinoma, and patients with FUS overexpression show a low survival rate. Increased abundance of m^6^A modification promotes FUS expression to enhance P53 and Gpx4 expression to promote the proliferation of prostate adenocarcinoma.^468^ m^6^A‐modified RNAs have been shown to prevent cytoplasmic TLS/FUS aggregation induced by hyperosmotic stress.^469^ SGs in U2OS cells induced by NaAsO_2_ treatment are enriched of m^6^A modified mRNA, and m^6^A binding of YTHDF proteins enhances SG formation.^17^ YTH domain family proteins, including YTHDF and YTHDC subtypes, have been reported to function in several types of cancers.^470^, ^471^ YTHDF2 has an essential regulatory role in various cancers (Figure 6),^472^, ^473^, ^474^ and acts as either an oncogene or tumor suppressor gene in different cancers depending on context.^475^ Generally, YTH domain family proteins play an essential role in tumor proliferation, invasion, migration, metabolism, and apoptosis.

In addition, it has been noted that SG formation can be beneficial for the survival of stressed cells, and persistent induction of SGs can cause cell death. SG assembly can be attenuated by circRNA–CREIT to activate the RACK1/MTK1 apoptosis signaling pathway in doxorubicin‐resistant triple‐negative breast cancer and combinatorial treatment of SG inhibitor ISRIB and doxorubicin would synergistically suppress the growth of doxorubicin‐resistant triple‐negative breast cancer.^476^ In another report, chemotherapy‐driven SGs have been shown to disassemble by glucocorticoids,^477^ suggesting that controlling SGs assembly can increase cancer cell chemosensitivity. However, a study has argued that m^6^A modification in mRNAs plays a minimal role, if any, in mRNA recruitment to SGs.^478^ Generally, m^6^A modification might modulate LLPS to regulate the development of cancer‐representative diseases under certain circumstances. However, more LLPS phenomena and their precise mechanism should be further exploited to benefit disease therapy.

## 7 IMPLICATIONS FOR DISEASE TREATMENT

### 7.1 Phase‐separated polymers can be used for diagnostics

Errors at any step of cell division might lead to aberrance in chromosome replication. Meanwhile, rearrangements and aneuploidy of the genome might occur in steps, such as chromosome condensation, breakdown of sister chromatids, and individualization of chromosomes as spatial segregates. Therefore, it is reasonable to apply phase‐separated polymers for disease diagnostics.

The expression of human Ki‐67 protein strictly correlates with cell proliferation and can be used as a value‐added marker of tumor cells.^479^ Only antigens can be localized in the nucleus during inter‐LLPS cell division, and proteins would be precisely localized on chromosomes during mitosis. The Ki‐67 protein presents during all active cell cycle phases (G1, S, G2, and mitosis). It is absent from resting cells (G0), making it an excellent marker for determining the so‐called growth fraction of a given cell population.^480^ Ki‐67 acts as a space, electrostatic charge barrier, or surfactant by phase‐separating individual mitotic chromosome arms.^481^ Generally, modulation by LLPS has the potential to enhance cancer diagnosis. To utilize LLPS behaviors for tumor diagnostics, real‐time tracking and mapping of the numerous endogenous proteins at once in living cells is critical. Recently, TransitID has been applied to unbiasedly map endogenous proteome trafficking among cytosol and different organelles in living cells, including nucleolus and SGs.^482^ Such a powerful approach for distinguishing protein populations even under the occurrence of LLPS can be conducive to developing precision medicine.

### 7.2 SGs can be targets for preventing disease progression and therapeutic resistance

**FIGURE 7:**
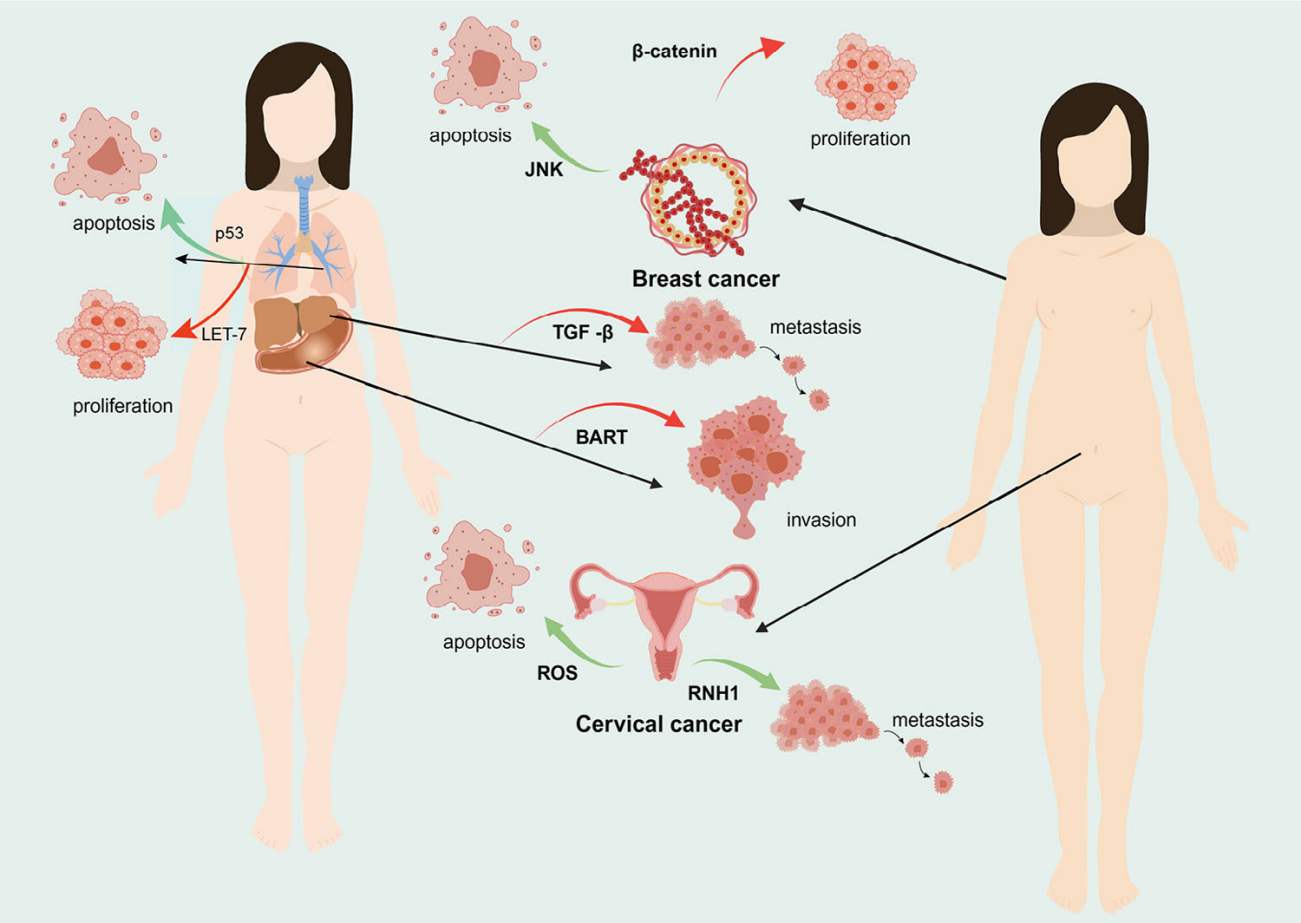
Role of SGs in typical cancer. Breast cancer: inhibition of apoptosis by downregulation of JNK versus promotion of proliferation, invasion, and metastasis by activating β‐catenin. Lung cancer: downregulation of p53 inhibits apoptosis and downregulation of LET‐7 promotes cell proliferation. Gastric cancer: upregulation of TGF‐β promotes invasion and metastasis. Pancreatic cancer: upward adjustment of BART facilitates invasion and metastasis. Cervical cancer: downregulation of ROS inhibits apoptosis and downregulation of RNH1 promotes invasion and metastasis.

Untranslated mRNPs bind over weak interactions and form cores or droplets that aggregate into SGs.^483^, ^484^ Normally, SGs are in dynamic equilibrium with polyribosomes, whereas pharmacological intervention or stress disrupts this equilibrium and blocks translation by inhibiting translation initiation.^484^, ^485^ It has been demonstrated that SGs regulate the body's behavior in various cancers, including lung, breast, pancreatic, and gastric cancer (Figure 7).^486^, ^487^, ^488^, ^489^, ^490^, ^491^, ^492^, ^493^ Particularly, SGs show strong antiapoptotic effects in pancreatic cancer.^494^ Breast cancer cell lines containing SGs exhibit lower sensitivity and less apoptosis when exposed to drugs.^495^ Generally, the formation of SGs would help the cancer cell to cope with the encountered stress to survive. Several studies have shown that some mRNA molecules contained in SGs can undergo a complete translation cycle and participate in and regulate various cellular biological processes.^496^ SGs‐related components are upregulated in multiple tumor cells, including G3BP1 and G3BP2. In lung cancer, G3BP1 has been shown to negatively regulate the p53 tumor suppressor gene by interacting with lncRNA to suppress apoptosis.^497^, ^498^ In non‐small cell lung cancer, a member of the TRIM protein family named MG53 has been revealed to modulate G3BP2 activity to inhibit SGs formation and suppress cancer progression.^499^ In addition, SGs may also be triggered in the high‐pressure tumor microenvironment, where SGs coordinate the cellular response to the harsh cellular environment. Targeting SGs by interfering with SGs formation or assembly and altering stress conditions can affect tumor progression and sensitize cancer cells to chemotherapeutic agents.^500^ Sodium arsenite induces production‐dependent SGs, participates in the regulation of apoptosis, protects cells in response to stress, inhibits apoptosis, and promotes cell survival.^499^, ^501^ Silvestrol‐induced production of nondependent SGs impairs cellular resistance to stress, reduces cell survival, and promotes apoptosis.^502^ Formation of SGs can contribute to the establishment of doxorubicin resistance, and circRNA–CREIT attenuates doxorubicin‐induced SGs formation via the PKR/eIF2α axis in triple‐negative breast cancer.^476^ In fact, many widely used chemotherapeutics have been found to induce SGs formation to drive cancer cells to obtain chemoresistance. SGs regulate tumor proliferation, apoptosis, invasion and drug resistance by participating in various tumor‐related signaling pathways.^503^, ^504^ mTOR is a critical signaling pathway that regulates cellular biological behavior, and studies have found that upregulation of mTOR promotes SGs formation in cancer cells.^501^, ^505^ Mutations in the RAS genes (K‐RAS, N‐RAS, and H‐RAS), the most frequently mutated genes in tumor cells, regulate the biosynthesis and catabolism of lipid signaling molecules, and contribute to the formation of SGs.^499^, ^506^ Therefore, engineering these pathways to destroy the aberrant formation of SGs and impaired SGs disassembly can contribute to eliminate the pathological phenomena in cancer.

As one of the most representative membraneless chambers, SGs also have an essential role in the pathophysiology of neurodegenerative diseases.^507^ SGs in neurons appear to be different from SGs that are generated rapidly after stress, possibly due to the presence of cytoplasmic factors that inhibit LLPS, such as posttranslational modifications, protein chaperones, specific cytoskeletal proteins, and so on.^485^ Neurodegenerative diseases with their slower pressure changes may also make the body's regulatory mechanisms more adaptable.^147^ Mutations in genes encoding pathologic aggregation proteins such as TDP43 and FUS lead to excessive accumulation of SGs in cells exposed to stress.^508^, ^509^ The adaptive response and accumulation of SGs to transient stress is fundamentally susceptible to aggregation‐based disease, and this high local concentration of increased amyloidogenic interactions would lead to the formation of persistent pathological oligomers. Targeted SGs could be used as a new option for treating neurodegenerative diseases. Studies have demonstrated the therapeutic benefit of inhibiting SGs‐associated RNA‐binding proteins. Knockdown of Atxn2 in an animal model of ALS overexpressing TDP43 increased the median lifespan of the animals by 80%,^510^ and inhibition of T1A1 prolonged survival of tau mice by 26%.^511^ In addition, targeting the neural pathways involved in SGs, including the mTOR pathway and the eIF2α pathway, is also considered as a potential therapeutic means.

### 7.3 RNA enzymes act as potential anticancer drug targets

RNA methylation is catalyzed by nucleoside methyltransferases, which act as critical proteins in a variety of RNA modifications (writers, erasers, and readers). Recently, developing anticancer epigenetic drugs has become a hot topic in cancer therapy research.^512^, ^513^ METTL3 is a well‐known m^6^A methyltransferase and is upregulated in multiple tumors and metastatic tissues. Elvitegravir, an anti‐HIV drug, directly targets METTL3 to prevent metastasis of esophageal squamous cell carcinoma.^514^ Actually, small‐molecule STM2457 has been designed to inhibit METTL3 and show antitumor ability against acute myeloid leukemia.^515^ Etrazepam, a thrombopoietin receptor agonist for the treatment of chronic immune thrombocytopenia and aplastic anemia, has been shown to act as a potential METTL3 inhibitor.^516^ In addition, renal inflammation and programmed cell death associated with the administration of cisplatin were attenuated in the presence of METTL3 silencing.^517^ METTL14 displays extremely high methyltransferase activity and is a crucial RNA‐binding scaffold that is essential in recognizing substrate RNAs. The SOCS family of proteins is involved in the negative feedback regulation of cytokine signaling, which disrupts cytokine downstream signaling and leads to the development of inflammatory and autoimmune diseases and malignant tumors.^518^ METTL14 can inhibit the proliferation and metastasis of colorectal cancer by targeting oncogenic lncRNA XIST.^519^ METTL14 is both a promoter and a tumor suppressor in cancer, and activators and inhibitors targeting METTL14 have recently been reported. METTL14 affects many biological processes and plays multiple roles in cancer,^520^ making it a potential drug target. ALKBH5, a demethylase of m^6^A, is involved in the induction of cancer immune escape and adaptive immune responses.^521^ Since ALKBH5 has both cancer‐inhibitory and cancer‐promoting roles, methods to induce and inhibit ALKBH5 are a viable therapeutic option.^522^ In addition, ALKBH5 demonstrated a regulatory role in renal impairment. ALKBH5 knockout mice exhibit less pathologic injury and better renal function, and the imposition of the ALKBH5 inhibitor IOX1 promotes Ccl28 modification to inhibit inflammatory cells.^523^ ALKBH5 has a high potential targeting capacity, and compounds, including small molecule modulators and protein hydrolysis‐targeted chimeras function at targeting the ALKBH5 regulatory mechanism.^524^, ^525^ Fibrillarin in 2ʹ‐O‐methylation has been reported to be a favorable target against tumors because it is an autoantibody for various cancers.^446^ Fibrillarin as a crucial constitutive protein affecting ribosome abundance and composition, offers significant possibilities for targeting key ribosome biogenesis components to reduce genotoxic activity in cancer cells.^526^ Investigating inhibitors targeting RNA modifications for the application of diseases is promising, but it is necessary to consider the role of LLPS during treatment as discussed above.

### 7.4 Targeting LLPS in drug design and delivery

Transcriptional control affects nearly all biological processes and events, including cancer initiation, proliferation, metastasis, recurrence, and drug resistance. LLPS has been observed to contribute to the maintenance of cellular homeostasis. Therefore, regulating the LLPS in forming SQSTM1/p62, NRF2, or FIP200 condensates would be conducive to recovering cellular homeostasis and preventing cancer progression. Furthermore, LLPS is also connected to cancer chemotherapeutic resistance, especially for involving with the formation of SGs. For example, cisplatin has been shown to suppress the formation of SGs.^527^ Meanwhile, future drug delivery system designs should focus on utilizing LLPS to enhance drug concentration and precision on targets. Based on some of the current applications of LLPS in drug delivery, precise delivery of drugs by RNA phase change to form polymers is possible.

Traditional drug development typically involves drugs that bind to and inhibit pathogenic targets, targeting proteins, and RNA via small molecule or RNA Phase modulators based on their propensity to phase separate.^255^, ^528^ Phase modulators can directly target RNA or proteins in IDR.^529^ In general, IDR are generally considered nondruggable therapeutic targets because they do not display stable, well‐defined binding sites. Depending on the screening of inhibitors of protein–protein interactions and phenotypic screening, a small molecule targeting the inherently disordered region of p53 interaction with MDM‐2 has been developed.^530^ YTHDF1 can bind to ribosomal proteins to facilitate translation of the mRNAs it targets,^531^ providing a direction for this protein to be used in targeted drug development.

Numerous anomalous phase‐separated drug screening and development platforms have been established to screen phase‐separated targeted drugs. In 2023, Wang et al.^532^ established DropScan, which could perform extensive screening and multidimensional analysis, identifying LY2835219, a condensate that solubilizes Ewing sarcoma fusions. Through this recognition of phase separation and the application of deep learning techniques, this platform could, in the future, screen suitable drugs in neurodegenerative, cardiovascular diseases, and so on.

Currently, there are also several applications of LLPS in drug delivery.^533^, ^534^ Regarding drug protection and degradability, Feng et al.^535^ used LLPS coupled with sodium alginate beads to enable drug release downstream in the gastrointestinal tract. The use of LLPS to deliver drugs, forming LLPS droplets to insulate their internal drug from physicochemical properties such as temperature, can lead to the design of LLPS systems capable of transporting active protein cascades to specific locations in human patients.^536^ In 2020, research validates the drug delivery potential of LLPS condensates by successfully delivering myoglobin to human mesenchymal stem cells using characteristics such as the noncytotoxic nature of the condensates and their spontaneous interaction with cell membranes.^537^ Nevertheless, LLPS that might impair or even reverse the destination is one of the prospects for designing and delivering cancer drugs.

## 8 CONCLUSION AND OUTLOOK

LLPS drives the assembly of multiple membraneless chambers of concentrated protein and RNA molecules through multivalent interactions. Multiple combinations between multivalent interactions driving LLPS transitions can lead to abnormal agglutination and amyloid formation and subsequently contribute to neurodegenerative disease, carcinogenesis, cancer progression, and therapeutic resistance.

Although the mechanism of LLPS in neurodegenerative diseases is not yet apparent, the link between abnormal phase separation and the pathology of several proteins has been well documented. LLPS concerning gene fusions and mutations can also affect cancer through many pathways, such as SPOP–DAXX bodies, PML vesicles and FET fusion proteins. Aberrant chromosome condensation generated by LLPS can induce tumorigenesis, and controlling the integrity of the nuclear microenvironment via LLPS can be tailored for cancer therapy. However, it should be noted that LLPS is just a biophysical phenomenon, and its functions in disease‐promoting or inhibiting are background dependent.

In addition to regulating nuclear structure, LLPS has a regulatory role in the transcription and translation. RNAs are a major constituent of several types of phase‐separated condensation, especially in RNA granules. Many condensates described to date contain mRNA, ncRNA, small molecules, and enzymes that can be used to modulate pathologic condensates or hard‐to‐administer targets. A variety of RNA modifications are either up‐ or downregulated in cancer and are involved in regulating the development of cancer cell fate. RNA modification is also involved in various diseases including cardiovascular disease, neurodevelopment and reproduction. Some of these modifications are observed to phase‐segregate in the formation of SGs. Regulatory RNA metabolizing enzymes with a large number of IDR regions are now available as oncogenic modulators and as new therapeutic targets. In recent years, the development of anticancer RNA therapeutics, including in vitro transcribed mRNA drugs, RNA vaccines, ASOs, and saRNA, has rapidly evolved.^538^, ^539^, ^540^

By discussing the close connection between LLPS, RNA modification, and diseases, it is believed that modulating RNA biology with LLPS as an alternative strategy for cancer treatment has a great outlook. However, the current research on LLPS mainly focus on proteins. Due to the scarcity of studies on LLPS with RNA modifications, it is impossible to draw arbitrary conclusions. More endeavors should be paid to enrich the understanding of the role of RNA, especially RNA modifications, in LLPS to contribute to disease treatment.

## AUTHOR CONTRIBUTIONS

Xinyue Zhang, Lin Yuan, Wanlu Zhang, Yi Zhang, Chunting Li, Qun Wu, and Yongye Huang participated in different parts of writing. Lin Yuan, Yongye Huang, and Min Wu composed and edited the manuscript. Xinyue Zhang and Wanlu Zhang illustrated the figure and artwork in consultation with coauthors. The article has received approval from all authors.

## CONFLICT OF INTEREST STATEMENT

The authors declare that they have no conflict of interest.

## ETHICS STATEMENT

Not applicable.

## Data Availability

Not applicable.
